# Elucidation of the Anti-Inflammatory Mechanisms of Bupleuri and Scutellariae Radix Using System Pharmacological Analyses

**DOI:** 10.1155/2017/3709874

**Published:** 2017-01-16

**Authors:** Xia Shen, Zhenyu Zhao, Hao Wang, Zihu Guo, Benxiang Hu, Gang Zhang

**Affiliations:** ^1^College of Pharmacy, Shaanxi University of Chinese Medicine, Xi'an, Shaanxi, China; ^2^Bioinformatics Center, College of Life Science, Northwest A&F University, Yangling, Shaanxi, China

## Abstract

*Objective.* This study was aimed at elucidating the molecular mechanisms underlying the anti-inflammatory effect of the combined application of Bupleuri Radix and Scutellariae Radix and explored the potential therapeutic efficacy of these two drugs on inflammation-related diseases.* Methods.* After searching the databases, we collected the active ingredients of Bupleuri Radix and Scutellariae Radix and calculated their oral bioavailability (OB) and drug-likeness (DL) based on the absorption-distribution-metabolism-elimination (ADME) model. In addition, we predicted the drug targets of the selected active components based on weighted ensemble similarity (WES) and used them to construct a drug-target network. Gene ontology (GO) analysis and KEGG mapper tools were performed on these predicted target genes.* Results.* We obtained 30 compounds from Bupleuri Radix and Scutellariae Radix of good quality as indicated by ADME assays, which possess potential pharmacological activity. These 30 ingredients have a total of 121 potential target genes, which are involved in 24 biological processes related to inflammation.* Conclusions.* Combined application of Bupleuri Radix and Scutellariae Radix was found not only to directly inhibit the synthesis and release of inflammatory cytokines, but also to have potential therapeutic effects against inflammation-induced pain. In addition, a combination therapy of these two drugs exhibited systemic treatment efficacy and provided a theoretical basis for the development of drugs against inflammatory diseases.

## 1. Introduction

Multiple chronic diseases are caused by inflammation, and the failure of resolution of inflammation increases the risk of development of such diseases [1]. Furthermore, inflammation is involved in many diseases, such as depression, bipolar disorder, atherosclerosis, and coronary artery disease [2–5].

In China, Traditional Chinese Medicine (TCM) has been used for the effective treatment of such diseases abovementioned for a long history. Amongst various TCM drugs, Bupleuri Radix and Scutellariae Radix are reported to possess anti-inflammation properties and similar patterns of therapeutic action against different diseases [6, 7]. In clinical application, prescription of Bupleuri Radix and Scutellariae Radix in combination is the most common therapy against inflammatory diseases. Saikosaponin constitutes the major anti-inflammatory components of Bupleuri Radix [8]. Baicalin and wogonoside, the main components in Scutellariae Radix, exhibit anti-inflammatory effects by suppressing the expression of IL-6, IL-8, and TNF-*α*, thus blocking the NF-*κ*B signaling pathway in HBE16 airway epithelial cells [9]. Although the therapeutic requirements against inflammatory diseases are different, a combination of Bupleuri Radix and Scutellariae Radix is frequently used with good therapeutic effects. However, the application of these two drugs against anti-inflammatory diseases is limited owing to the lack of information regarding their mechanisms of action.

**Figure 1:**
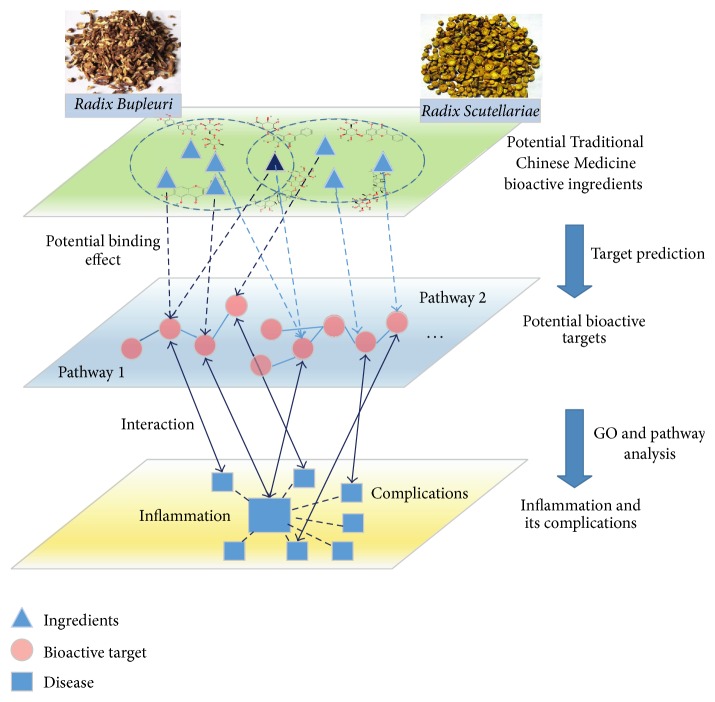
The brief workflow of system pharmacological analyses in searching* Bupleuri-Scutellariae Radix* anti-inflammation mechanism.

Systems pharmacology based research strategy has been widely used in many TCM herbal medicines and formulas researching, such as* Folium Eriobotryae* [10], licorice [11], and Danggui-Shaoyao-San decoction [12], which is helpful for the understanding of the molecular mechanism of TCM treatment from a microcosmic view. It mainly contains ADME screening, interactions network analysis, and pathway analysis [13]. Therefore, in order to broaden our knowledge on Bupleuri Radix and Scutellariae Radix, we analyzed the ingredients of these two drugs using systems pharmacology. And we predicted the target genes of the active components and traced the inflammatory pathways related to these targets. Our study explored the cellular mechanisms underlying the anti-inflammatory activities of these two drugs, providing a molecular basis for the treatment of various diseases and their complications using Traditional Chinese Medicine (TCM), as shown in our brief workflow (Figure 1).

## 2. Materials and Methods

### 2.1. Materials

The ingredients structures of two herbal medicines were collected from Traditional Chinese Medicines for Systems Pharmacology Database and Analysis Platform (TCMSP http://lsp.nwsuaf.edu.cn/tcmsp.php) and Traditional Chinese Medicines Integrated Database (TCMID http://www.megabionet.org/tcmid/) and supplement the lacking ingredients by the literature from NCBI PubChem database (https://pubchem.ncbi.nlm.nih.gov/) and CNKI database (http://www.cnki.net/).

### 2.2. Active Ingredients Screening

Previous prediction is very necessary for the drug development process. Accurate identification of the active ingredients from herbal medicines is a basic step to assess the therapeutic mechanism of herbal medicines. And it is helpful for understanding the molecular mechanisms through pharmacokinetic characteristics research. Thus, ADME screening method and a series of pharmacokinetic parameters containing oral bioavailability (OB) and drug-likeness (DL) prediction were employed as our first step. Oral bioavailability is usually used to determine that the orally administered drugs could overcome several barriers and delivery into systemic circulation. A computer model OBioavail which integrates with the metabolism (cytochromes P450 3A4 and 2D6) and transport (P-glycoprotein) information is applying to predict the OB value of herbal ingredients [14]. In our present work, we chose the ingredients with OB ≥ 30%. Drug-likeness (DL) prediction allowed us to remove the ingredients deemed to be chemically unsuitable for drug here. It can be deduced that the absorption, distribution, metabolism, and excretion of the herbal medicine ingredients in human body are affected. This model was based on Tanimoto similarity defined as

(1) $$\begin{array}{c} T\left(\;A,B\;\right)=\frac{A\cdot B}{{\left|A\right|}^{2}+{\left|B\right|}^{2}-A\cdot B}. \end{array}$$

In the formula above, “*A*” represents the ingredients from two herbal medicines, and “*B*” represents the average drug-likeness index of all molecules in DrugBank database based on Dragon software descriptors. The DL value represents the possibility that the compound may possess certain biological properties. In our work, we choose the ingredients with suitable DL (DL ≥ 0.18), because the average DL index of DrugBank molecules is 0.18.

### 2.3. Potential Targets Fishing

In order to identify the molecular targets, a novel weighted ensemble similarity (WES) algorithm was employed to predict the potential treatment targets of 60 potential ingredients [15]. This model was built on a large data set involving 98,327 drug-target relations based on BindingDB (http://www.bindingdb.org/bind/index.jsp), DrugBank (http://www.drugbank.ca/), PDB database (http://www.rcsb.org/pdb/), and GoPubMed (http://www.ncbi.nlm.nih.gov/). And the algorithm mainly contains three parts: (1) the first is identifying the key ligand structural and physicochemical features by CDK and Dragon software; (2) in order to improve the accuracy, the overall similarities were converted into the size-bias-free normalized values to eliminate the relevant similarities from random; (3) finally, Bayesian network was used to predict the ensemble similarities (*Z* score). Then we chose the targets, which score greater than 5, as the potential targets.

Next, we standard the related targets and their related genes by using the BLAST tool in Uniprot database (http://www.uniprot.org/blast/). Then we collected the inflammatory diseases and potential diseases from CTD (http://ctdbase.org/), TTD (http://bidd.nus.edu.sg/group/cjttd/), and PharmGKB database (https://www.pharmgkb.org/).

### 2.4. Network Construction and Pathway Analysis

TCM prescriptions are considered as multicomponent therapeutics like multiple herbal medicines ingredients interacting with multiple targets. In order to explore the molecular mechanism of Bupleuri Radix and Scutellariae Radix for inflammation and complication diseases, we mapped the ingredients, targets, and potential diseases relevant to inflammation.

And the ClueGO, a plugin from Cytoscape, was utilized to interpret the related gene biology processes. Meanwhile, we used the KEGG Mapper analysis tool (http://www.genome.jp/kegg/tool/map_pathway2.html) to construct the pathways of these genes relevant to inflammatory diseases.

In order to find a disease with a potential comechanism with inflammation, the related disease information based on the potential bioactive was obtained from CTD database (http://ctdbase.org/), PharmGKB (https://www.pharmgkb.org/index.jsp), and TTD database (http://bidd.nus.edu.sg/group/cjttd/). And we classified these related diseases by NIH MeSH (https://meshb.nlm.nih.gov/#/fieldSearch). Finally, a target-disease-MeSH network was built by Cytoscape software.

## 3. Results and Discussion

### 3.1. Active Natural Ingredients

Based on the administration-distribution-metabolism-elimination (ADME) model, 30 active constituents out of 99 (Table S1 in Supplementary Material available online at https://doi.org/10.1155/2017/3709874) were selected (OB > 30%, DL > 0.18), as shown in Table 1. These 30 natural products all possess high oral bioavailability and drug-likeness. Among them, 21 components were discovered in Scutellariae Radix and 9 in Bupleuri Radix. Interestingly, this finding, consistent with the crucial nature of the function performed by Scutellariae Radix (clearing away heat and removing toxins), suggests that Scutellariae Radix would be more commonly used against inflammatory diseases than Bupleuri Radix [2].

Not surprisingly, most of the selected active ingredients are directly or indirectly related to inflammation. Several ingredients including saikogenin G, stigmasterol, salvigenin, ganhuangenin, and norwogonin have a direct therapeutic effect against inflammatory diseases. In a previous study, Saikogenin G was demonstrated to exhibit an anti-inflammatory activity against carrageenan produced plantar edema in rats [16]. Stigmasterol has exhibited anti-inflammatory and immunomodulatory effects through the downregulation of proinflammatory cytokines; moreover, it has been shown to inhibit herpes simplex virus replication in nervous cells in vitro [17, 18]. Devi and Periyanayagam found that Salvigenin derived from* Plectranthus amboinicus* exerted anti-inflammatory effects through human red blood cell (HRBC) membrane stabilization [19]. Bo et al. found that ganhuangenin obtained from Scutellariae Radix inhibited the release of histamine and leukotriene B4, thus inducing antioxidation and anti-inflammatory effects [20]. Norwogonin, an active component of Scutellariae Radix, selectively suppresses the activity of COX-1, COX-2, and 5-LOX and exhibits anti-inflammatory activity in arachidonic acid-induced mouse auricular edema. Skullcapflavone II, a potential bradykinin antagonist, was reported to relieve the inflammation in mouse asthma via regulation of the TGF-*β*1/Smad signaling pathway [21]. More importantly, Scutellariae Radix and Bupleuri Radix modulate immune function. Campesterol is an active ingredient acting as an anti-inflammatory and immunoregulatory compound [22]. Campesterol, a common type of plant sterol, exhibits both anti-inflammatory activity and immunomodulatory effects in Jurkat T cells through IL-2-mediated cAMP modulation and/or a calcium/calcineurin-independent pathway [23].

Inflammation is a common pathological process in many diseases. This means that natural products against inflammation have a wide range of therapeutic mechanisms of action. For instance, chrysin exhibits antitumor, antioxidation, and anti-inflammation activities; Bae et al. found that chrysin suppressed systemic anaphylaxis, histamine release, and IgE-mediated local anaphylaxis in mast cells. Therefore, it can be concluded that chrysin modulates allergic inflammation better than sodium cromoglycate (Intal) [24]. Shin et al. found that chrysin also exhibited anti-inflammatory effects by inhibiting the activation of NF-*κ*B [25]. Meanwhile, chrysin could suppress NF-*κ*B activity through the sensitization of TNF-*α* by inducing tumor cell apoptosis [26]. Therefore, chrysin may be developed into a drug for the treatment of tumors concomitant with inflammation. Saikosaponin C, a major component in Bupleuri Radix, exhibited good ADME properties in our study. Moreover, it has been reported to suppress caspase-3 activity and caspase-3-mediated FAK degradation to prevent LPS-induced cell injury and apoptosis [27]. In addition, saikosaponin C inhibits the release of amyloid beta proteins 1-40 and 1-42 as well as abnormally phosphorylated tau, suggesting that saikosaponin C could be applied in the treatment of Alzheimer's disease [28].

### 3.2. Drug-Target Network Analyses

**Figure 2:**
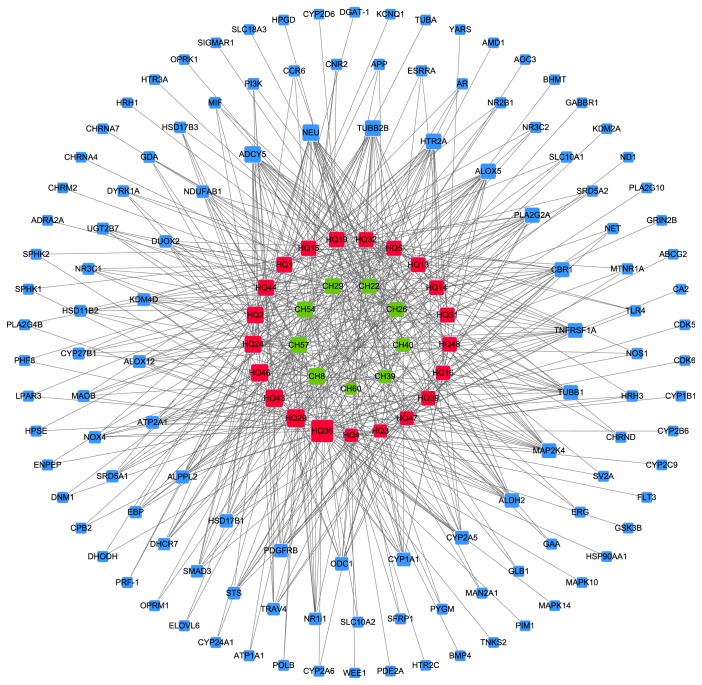
Bupleuri Radix-Scutellariae Radix active ingredients and potential drug targets. Blue: target gene ID; green: active ingredient in Bupleuri Radix; red: active ingredient in Scutellariae Radix. Node size indicates the degree in the network—bigger nodes represent more target genes and smaller nodes indicated fewer targets.

To elucidate the molecular mechanisms underlying the anti-inflammatory properties of the two drugs, we predicted the drug targets of the 30 selected active components based on weighted ensemble similarity (WES) analysis. The results showed that these 30 ingredients had 121 potential targets (Table S2). Moreover, we established a drug-target network using Cytoscape 3.2.0 software, and the target gene ID, as given in Uniprot, is shown in Figure 2.

The drug targets with a high degree and multiple ingredients were further identified and summarized. Some of these drug targets were directly correlated with cytokine synthesis and release, such as adenylate cyclase type V (ADCY5). In a model of neurogenic inflammation in mouse ear, ADCY5 was found to play a critical role in sensory neuropeptide release and neurogenic inflammation [29]. Katoh et al. discovered the crucial role of sialidase (Neu) in the hyaluronan receptor function of CD44 in T helper type 2-mediated airway inflammation in a murine acute asthmatic model [30]. It is well known that 5-lipoxygenase (ALOX5) is a key enzyme in the biosynthesis of leukotrienes from arachidonic acid in inflammatory and allergic processes. ALOX5 is therefore critical in multiple inflammatory diseases [31]. Fibrosis is a common complication in inflammation. PLA2G2A gene expression is elevated in ulcerative colitis and Crohn's disease, suggesting that this gene is important in inflammation in these two diseases [32]. Toll-like receptor (TLR) pathway is important in inflammation and critical in the migration of pancreatic cancer cells. MAP2K4 is a key protein involved in the migratory process in cancer cells [33]. TNFRSF1A and TLR4 are reported to be implicated in inflammation, and TNFRSF1A is involved in the inflammatory response syndrome [34, 35].

Another kind of drug targets is involved in complications in inflammatory diseases. The neurotransmitter 5-hydroxytryptamine (5-HT) plays an important role in immune responses and inflammatory diseases, such as inflammatory bowel disease, airway inflammation, and rheumatoid arthritis. HTR2A regulates the expression of 5-HT and is closely associated with inflammatory processes [36]. ALDH2 is involved in oxidative stress and inflammation in diabetes [37]. It can thus clearly be seen that the predicted drug targets in our study are associated with inflammation in an either direct or indirect manner.

From the network, we observed interaction between the 30 components and their predicted drug targets. In addition, the degrees of chrysin (HQ36), norwogonin (HQ02), saikosaponin C (CH54), and thymonin (CH40) were 39, 21, 18, and 11, respectively. These active components interacted with their predicted drug targets, such as ADCY5, NEU, ALOX5, HTR2A, MAP2K4, and TLR4; these drug targets are critical in the inflammatory process, and multiple components exhibited synergistic effects through the concurrent regulation of inflammation-related targets. For instance, chrysin interacted not only with the genes directly related to inflammation, such as ALOX5, TLR4, MAPL10, MAPK14, and MAPK2K4, but also with genes indirectly related to inflammation, such as HTR2A, HSP90AA1, and ADCY5. Norwogonin had 21 targets including ALOX5 and MAP2K4, which are directly involved in inflammation. Thymonin interacted with the inflammation-related targets Alox5, Alox12 TNFRSF1A, and PDGFRB. PDGFRB plays a key role in inflammatory and noninflammatory breast cancer [38]. Saikosaponin C (SSc), a major component of* Bupleuri Radix*, interacted with CNR2. Activation of CNR2 inhibits the release of lymphokine and angiogenic factors, thus influencing the inflammation process and carcinogenesis [39]. Meanwhile, the SSc target SFRP1 is closely related to cancers such as prostate cancer [40], bladder cancer [40], and acute myeloid leukemia [41]. Moreover, these active ingredients exhibited systemic therapeutic efficacy by acting on targets indirectly involved in inflammation and its complications. In this way, the active ingredients of Bupleuri Radix and Scutellariae Radix exhibit anti-inflammatory effects by regulating not only those targets critical for inflammation but also those targets indirectly involved in inflammation and its complications.

### 3.3. GO and KEGG Pathway Analysis of the Inflammation-Related Drug Targets

To elucidate the molecular mechanisms underlying the anti-inflammatory effects of the two drugs, we performed gene set enrichment analysis using the Cytoscape plugin “ClueGO.” As a result, we obtained 24 significant biological processes (*P* < 0.05). To better show the corresponding targets in pathway, we collate the targets associated with each pathway into the table, as shown in Table S3. In this section, we mainly aimed at the inflammation-related pathways and try to dig out underlying disease with the same pathogenesis.

**Figure 3:**
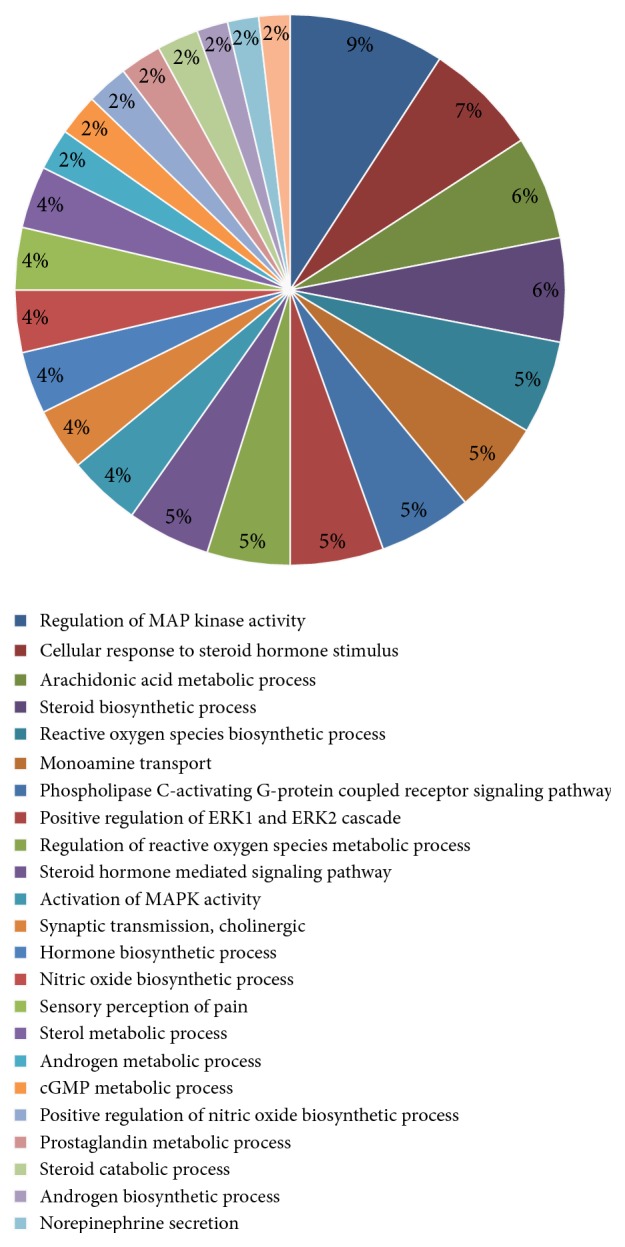
The gene count of inflammation-related gene ontology (GO) term classification.

As shown in Figure 3, we found that a number of the targets were involved in the classic inflammatory pathways and in transmitting mediators of inflammation. The other targets were associated with the complications and symptoms of inflammation. Some drug targets are related to MAPK activation, such as MAP2K4, TLR4, MAPK10, MAPK14, NOX4, ADAR2A, CHRNA7, ERBB2, FLT3, HTR2A, and LPAR3. Among these genes, TLR4 regulates LPS-induced inflammation through modulation of the P38 MAPK signaling pathway [42]. Others are involved in the metabolism of arachidonic acid, such as ALOX12, PLA2G10, PLA2G4B, CYP1B1, and CYP2A6. ALOX12 regulates the concentration of arachidonic acid in the peripheral blood via lipoxygenase [43]. Steroids exhibit anti-inflammatory effects through inhibition of the release of arachidonic acid and synthesis of prostaglandin [44]. Therefore, related target genes were also listed, such as ATP1A1, CYP27B1, DHCR7, EBP, GLB1, HSD11B2, HSD17B1, HSD17B3, SRD5A1, and SRD5A2.

**Figure 4:**
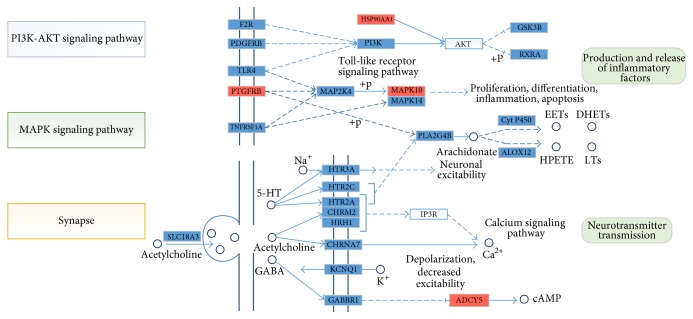
KEGG pathways of inflammation-related bioactive targets. Blue block: the target can be affected by the ingredients from two herbal medicines; red block: cancer-related targets in the KEGG database; white block: key target in the related pathway, but having no binding effect with our herbal ingredients; small circle: chemical metabolites in pathway.

In order to clearly explain the two Chinese medicines' corresponding pathway, we built a pathway figure by KEGG Mapper analysis tool (as shown in Figure 4). We have found that Bupleuri Radix and Scutellariae Radix may be beneficial for the treatment of inflammation-induced pain. NO is an inflammatory cytokine released by multiple cells. GO analysis showed that NO was closely related to CYP1B1, HSP90AA1, NOS1, OPRM1, and SMAD3. NOS1 in particular is a key enzyme in NO synthesis [45]. For example, we found target genes involved in the opioid receptor pathway (OPRK1, OPRM1, and SIGMAR1) and in pathways relating to synaptic release of neurotransmitters and hydroxytryptamine (HT) transmission (HTR2A and CHRNA4). Targeting the opioid receptor is a common strategy to achieve pain relief [46]. HTR2A, which is important for 5-HT transmission and nerve conduction, presents elevated expression levels in the punctured rat disc [47].

Combining with the information above, we speculated that the active ingredients in Bupleuri Radix and Scutellariae Radix not only act on the MAPK pathway, NO synthesis pathway, and the arachidonic acid pathway to directly regulate the synthesis and release of inflammatory cytokines, but also affect synaptic release of neurotransmitters in order to achieve pain relief. As a result, these two drugs exhibit systemic anti-inflammatory activity.

**Figure 5:**
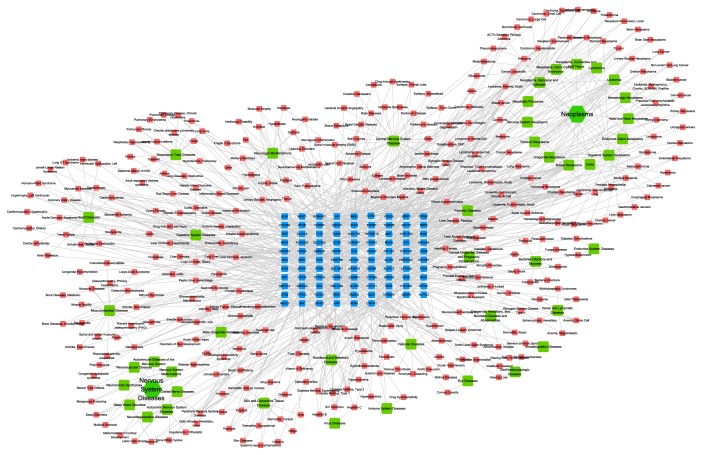
Target-disease-MeSH network. Blue node: the potential bioactive targets. Red node: potentially relevant diseases from CTD, TTD, and PharmGKB databases. Green node: MeSH classifications of potentially relevant disease.

Interestingly, according to the targets from above and disease database mining, 344 diseases in 45 classifications were related to 96 targets (Figure 5), which are mostly neoplasms-related diseases (see the details in Table S4). In summary, common targets of these diseases are mostly binding by the ingredients from herbal medicines of Bupleuri Radix and Scutellariae Radix. Thus, these two herbal medicines may relieve complications and secondary disease of inflammatory diseases and treat them in common targets through some common signaling pathways. This may indirectly prove that these natural ingredients can be developed as the core drug of these diseases. Thus, our result provides new information and methodology reference on clinical using for these two herbal medicines and the prodrug discovery of their natural ingredients.

## 4. Conclusion

Inflammation is a complex pathologic process usually accompanying other diseases, and long-term inflammation increases the risk of cancer [48, 49]. This complexity means that resolution of inflammation is not enough to eradicate diseases. It is critical to systemically treat both inflammation and its complications.

Previously, antipathogen drugs including cephalosporins, aminoglycosides, and penicillin were often used to treat inflammation, leading to severe renal/nerve toxicity and amino glycosides-induced allergic reactions in some patients. Moreover, single-target drugs such as COX-2 inhibitor are limited in clinical application. TCM has multiple components and thus exhibits diverse pharmaceutical activities. Therefore, it is practical to develop novel anti-inflammation drugs from these and other natural products.

In the present study, we first analyzed the components of the selected TCM, Bupleuri Radix and Scutellariae Radix, using a systems pharmacology computer model and database mining technology. Second, the molecular mechanisms underlying the anti-inflammation of the active ingredients were elucidated. Our study revealed that TCM has multiple components and multiple targets. The active ingredients of TCM interacted with key targets to inhibit the release of inflammatory cytokines and promote the production of immune cytokines in order to systemically improve the body's immunity. We selected 30 active ingredients with potential anti-inflammatory activity. Among them, several ingredients including saikogenin G, stigmasterol, salvigenin, ganhuangenin, and norwogonin are directly involved in the production and release of inflammatory cytokines. Campesterol, chrysin, and saikosaponin C not only are related to the production and release of inflammatory cytokines, but also exhibit activity against cancer-related inflammation. Thus, these ingredients could possibly be developed into therapeutics for treating inflammation and associated tumors. In addition, the abovementioned ingredients interacted with TLR4, ALOX5, ALOX12, and MAPK10, all of which are critical for the inflammatory process. Disruption of these targets affected the biosynthesis of leukotrienes from arachidonic acid. Interestingly, the selected components potentially interacted with OPRK1 and HTR2A, two target genes involved in pain, suggesting that TCM could relieve both inflammation and its common complication: pain. The multiple components and targets of TCM endow it with diverse pharmacological effects. However, the inflammation-related targets have not been adequately analyzed because of limited specific investigation of these genes. We will further explore the mechanism of action of anti-inflammatory effects of various TCM products and provide a rational basis for drug development in the future.

## Supplementary Material

The interaction of compounds and their biological targets.

## Figures and Tables

**Table 1:** Potential active constituents in *Bupleuri Radix* and *Scutellariae Radix*.

ID	Ingredient name	OB	DL
CH08	Stigmasterol	43.82985	0.75664
CH22	Areapillin	55.14803	0.41394
CH26	Octalupine	47.82225	0.27864
CH29	Saikogenin G	51.83940	0.63197
CH39	Sainfuran	81.60749	0.23333
CH40	Thymonin	43.16284	0.40714
CH54	Saikosaponin c_qt	30.51828	0.63193
CH57	*α*-Spinasterol	42.97937	0.75693
CH60	Cubebin	57.12813	0.63980
HQ01	Campesterol	35.02838	0.71579
HQ02	Norwogonin	40.44827	0.20723
HQ03	5,2′-Dihydroxy-6,7,8-trimethoxyflavone	30.07322	0.35463
HQ04	Coptisine	30.40885	0.85647
HQ05	Supraene	33.54594	0.42162
HQ13	Carthamidin	40.28190	0.24188
HQ14	Dihydrobaicalin	41.53938	0.20722
HQ15	Salvigenin	53.87782	0.33279
HQ16	Ganhuangenin	93.43294	0.37375
HQ19	5,7,2′,6′-Tetrahydroxyflavone	35.42827	0.24383
HQ24	5,7,4′-Trihydroxy-8-methoxyflavone	34.76242	0.26666
HQ29	11,13-Eicosadienoic acid	39.27534	0.22891
HQ31	5,7,4′-Trihydroxy-6-methoxyflavanone	37.00241	0.26833
HQ32	5,2′-Dihydroxy-7,8,6′-trimethoxyflavone	38.39282	0.36629
HQ36	Chrysin	48.03082	0.18140
HQ39	Dihydrooroxylin A	46.37778	0.23057
HQ43	Oroxylin A	45.40775	0.23231
HQ44	Rivularin	43.74214	0.36628
HQ46	Skullcapflavone I	51.70113	0.29148
HQ47	Skullcapflavone II	43.90662	0.43793
HQ48	Tenaxin I	32.77480	0.35463

## References

[1] Luo B., Wang J., Liu Z. (2016). Phagocyte respiratory burst activates macrophage erythropoietin signalling to promote acute inflammation resolution. *Nature Communications*.

[2] Capuron L., Lasselin J., Castanon N. (2017). Role of adiposity-driven inflammation in depressive morbidity. *Neuropsychopharmacology*.

[3] Alie N., Eldib M., Fayad Z. A. (2014). Inflammation, atherosclerosis, and coronary artery disease: PET/CT for the evaluation of atherosclerosis and inflammation. *Clinical Medicine Insights: Cardiology*.

[4] Berk M., Kapczinski F., Andreazza A. C. (2011). Pathways underlying neuroprogression in bipolar disorder: focus on inflammation, oxidative stress and neurotrophic factors. *Neuroscience and Biobehavioral Reviews*.

[5] Ridker P. M., Hennekens C. H., Buring J. E., Rifai N. (2000). C-reactive protein and other markers of inflammation in the prediction of cardiovascular disease in women. *The New England Journal of Medicine*.

[6] Jiang W.-Y. (2005). Therapeutic wisdom in traditional Chinese medicine: a perspective from modern science. *Trends in Pharmacological Sciences*.

[7] Yoon S.-B., Lee Y.-J., Park S. K. (2009). Anti-inflammatory effects of *Scutellaria baicalensis* water extract on LPS-activated RAW 264.7 macrophages. *Journal of Ethnopharmacology*.

[8] Ma Y., Bao Y., Wang S. (2016). Anti-inflammation effects and potential mechanism of saikosaponins by regulating nicotinate and nicotinamide metabolism and arachidonic acid metabolism. *Inflammation*.

[9] Dong S.-J., Zhong Y.-Q., Lu W.-T., Li G.-H., Jiang H.-L., Mao B. (2015). Baicalin inhibits lipopolysaccharide-induced inflammation through signaling NF-*κ*B pathway in HBE16 airway epithelial cells. *Inflammation*.

[10] Zhang J., Li Y., Chen S.-S. (2015). Systems pharmacology dissection of the anti-inflammatory mechanism for the medicinal herb *Folium Eriobotryae*. *International Journal of Molecular Sciences*.

[11] Liu H., Wang J., Zhou W., Wang Y., Yang L. (2013). Systems approaches and polypharmacology for drug discovery from herbal medicines: an example using licorice. *Journal of Ethnopharmacology*.

[12] Luo Y., Wang Q., Zhang Y. (2016). A systems pharmacology approach to decipher the mechanism of danggui-shaoyao-san decoction for the treatment of neurodegenerative diseases. *Journal of Ethnopharmacology*.

[13] Huang C., Zheng C., Li Y., Wang Y., Lu A., Yang L. (2013). Systems pharmacology in drug discovery and therapeutic insight for herbal medicines. *Briefings in Bioinformatics*.

[14] Xu X., Zhang W., Huang C. (2012). A novel chemometric method for the prediction of human oral bioavailability. *International Journal of Molecular Sciences*.

[15] Zheng C., Guo Z., Huang C. (2015). Large-scale direct targeting for drug repositioning and discovery. *Scientific Reports*.

[16] Utrilla M. P., Zarzuelo A., Risco S., Ocete M. A., Jimenez J., Gamez M. J. (1991). Isolation of a saikosaponin responsible for the antiinflammatory activity of *Bupleurum gibraltaricum* Lam. root extract. *Phytotherapy Research*.

[17] Villa-de la Torre F., Kinscherf R., Bonaterra G. (2016). Anti-inflammatory and immunomodulatory effects of *Critonia aromatisans* leaves: downregulation of pro-inflammatory cytokines. *Journal of Ethnopharmacology*.

[18] Petrera E., Níttolo A. G., Alché L. E. (2014). Antiviral action of synthetic stigmasterol derivatives on herpes simplex virus replication in nervous cells in vitro. *BioMed Research International*.

[19] Devi K. N., Periyanayagam K. (2010). In vitro anti-inflammatory activity of *Plectranthus amboinicus* (Lour) Spreng by HRBC membrane stabilization. *International Journal of Pharmaceutical Sciences and Research*.

[20] Lim B. O. (2002). Effect of ganhuangenin obtained from *Scutellaria radix* on the chemical mediator production of peritoneal exudate cells and immunoglobulin E level of mesenteric lymph node lymphocytes in Sprague-Dawley rats. *Phytotherapy Research*.

[21] Jang H.-Y., Ahn K.-S., Park M.-J., Kwon O.-K., Lee H.-K., Oh S.-R. (2012). Skullcapflavone II inhibits ovalbumin-induced airway inflammation in a mouse model of asthma. *International Immunopharmacology*.

[22] Navarro A., De las Heras B., Villar A. (2001). Anti-inflammatory and immunomodulating properties of a sterol fraction from *Sideritis foetens* CLEM. *Biological and Pharmaceutical Bulletin*.

[23] Aherne S. A., O'Brien N. M. (2008). Modulation of cytokine production by plant sterols in stimulated human Jurkat T cells. *Molecular Nutrition and Food Research*.

[24] Bae Y., Lee S., Kim S.-H. (2011). Chrysin suppresses mast cell-mediated allergic inflammation: involvement of calcium, caspase-1 and nuclear factor-*κ*B. *Toxicology and Applied Pharmacology*.

[25] Shin E. K., Kwon H.-S., Kim Y. H., Shin H.-K., Kim J.-K. (2009). Chrysin, a natural flavone, improves murine inflammatory bowel diseases. *Biochemical and Biophysical Research Communications*.

[26] Li X., Huang Q., Ong C.-N., Yang X.-F., Shen H.-M. (2010). Chrysin sensitizes tumor necrosis factor-*α*-induced apoptosis in human tumor cells via suppression of nuclear factor-kappaB. *Cancer Letters*.

[27] Lee T. H., Chang J., Kim B. M. (2014). Saikosaponin C inhibits lipopolysaccharide-induced apoptosis by suppressing caspase-3 activation and subsequent degradation of focal adhesion kinase in human umbilical vein endothelial cells. *Biochemical and Biophysical Research Communications*.

[28] Lee T. H., Park S., You M.-H., Lim J.-H., Min S.-H., Kim B. M. (2016). A potential therapeutic effect of saikosaponin C as a novel dual-target anti-Alzheimer agent. *Journal of Neurochemistry*.

[29] Németh J., ReglÖdi D., Pozsgai G. (2006). Effect of pituitary adenylate cyclase activating polypeptide-38 on sensory neuropeptide release and neurogenic inflammation in rats and mice. *Neuroscience*.

[30] Katoh S., Maeda S., Fukuoka H. (2010). A crucial role of sialidase Neu1 in hyaluronan receptor function of CD44 in T helper type 2-mediated airway inflammation of murine acute asthmatic model. *Clinical and Experimental Immunology*.

[31] Steinhilber D. (1999). 5-lipoxygenase: a target for antiinflammatory drugs revisited. *Current Medicinal Chemistry*.

[32] Wu F., Chakravarti S. (2007). Differential expression of inflammatory and fibrogenic genes and their regulation by NF-*κ*B inhibition in a mouse model of chronic colitis. *Journal of Immunology*.

[33] Liu J., Xu D., Wang Q., Zheng D., Jiang X., Xu L. (2014). LPS induced miR-181a promotes pancreatic cancer cell migration via targeting PTEN and MAP2K4. *Digestive Diseases and Sciences*.

[34] Bank S., Skytt Andersen P., Burisch J. (2014). Polymorphisms in the inflammatory pathway genes TLR2, TLR4, TLR9, LY96, NFKBIA, NFKB1, TNFA, TNFRSF1A, IL6R, IL10, IL23R, PTPN22, and PPARG are associated with susceptibility of inflammatory bowel disease in a Danish cohort. *PLoS ONE*.

[35] Borghini S., Ferrera D., Prigione I. (2016). Gene expression profile in TNF receptor-associated periodic syndrome reveals constitutively enhanced pathways and new players in the underlying inflammation. *Clinical and Experimental Rheumatology*.

[36] Shajib M. S., Khan W. I. (2015). The role of serotonin and its receptors in activation of immune responses and inflammation. *Acta Physiologica*.

[37] Wang H.-J., Kang P.-F., Wu W.-J. (2013). Changes in cardiac mitochondrial aldehyde dehydrogenase 2 activity in relation to oxidative stress and inflammatory injury in diabetic rats. *Molecular Medicine Reports*.

[38] Chai F., Liang Y., Zhang F., Wang M., Zhong L., Jiang J. (2016). Systematically identify key genes in inflammatory and non-inflammatory breast cancer. *Gene*.

[39] Staiano R. I., Loffredo S., Borriello F. (2016). Human lung-resident macrophages express CB1 and CB2 receptors whose activation inhibits the release of angiogenic and lymphangiogenic factors. *Journal of Leukocyte Biology*.

[40] García-Tobilla P., Solórzano S. R., Salido-Guadarrama I. (2016). *SFRP1* repression in prostate cancer is triggered by two different epigenetic mechanisms. *Gene*.

[41] An C., Guo H., Wen X.-M. (2015). Clinical significance of reduced SFRP1 expression in acute myeloid leukemia. *Leukemia and Lymphoma*.

[42] Li Q., Bao F., Zhi D. (2016). Lipopolysaccharide induces SBD-1 expression via the P38 MAPK signaling pathway in ovine oviduct epithelial cells. *Lipids in Health and Disease*.

[43] Berthelot C. C., Kamita S. G., Sacchi R. (2015). Changes in PTGS1 and ALOX12 gene expression in peripheral blood mononuclear cells are associated with changes in arachidonic acid, oxylipins, and oxylipin/fatty acid ratios in response to omega-3 fatty acid supplementation. *PLoS ONE*.

[44] Floman Y., Zor U. (1976). Mechanism of steroid action in inflammation: inhibition of prostaglandin synthesis and release. *Prostaglandins*.

[45] Coleman J. W. (2001). Nitric oxide in immunity and inflammation. *International Immunopharmacology*.

[46] Spetea M., Asim M. F., Wolber G., Schmidhammer H. (2013). The opioid receptor and ligands acting at the *μ* opioid receptor, as therapeutics and potential therapeutics. *Current Pharmaceutical Design*.

[47] Fujioka Y., Stahlberg A., Ochi M., Olmarker K. (2016). Expression of inflammation/pain-related genes in the dorsal root ganglion following disc puncture in rats. *Journal of Orthopaedic Surgery*.

[48] Roseweir A. K., Powell A. G. M. T., Bennett L. (2016). Relationship between tumour PTEN/Akt/COX-2 expression, inflammatory response and survival in patients with colorectal cancer. *Oncotarget*.

[49] Benedetti I., Bettin A., Reyes N. (2016). Inflammation and focal atrophy in prostate needle biopsy cores and association to prostatic adenocarcinoma. *Annals of Diagnostic Pathology*.

