# Supplementary material for: Elucidation of the Anti-Inflammatory Mechanisms of Bupleuri and Scutellariae Radix Using System Pharmacological Analyses
Source: Mediators Inflamm. 2017 Jan 16;2017:3709874. doi: 10.1155/2017/3709874 (PMC5278517; doi:10.1155/2017/3709874)
Supplement: Supplementary file 1 — The interaction of compounds and their biological targets. [file 3709874.f1.docx]

Supplementary files:

Table S1 Potential active constituents in Bupleuri Radix and Scutellaria Radix

| ID | OB | DL | name |
| --- | --- | --- | --- |
| CH10 | 41.52886 | 0.030035 | thymol |
| CH11 | 14.0498 | 0.43379 | Pregnenolone |
| CH12 | 14.15435 | 0.76678 | Pregnenolone |
| CH14 | 2.004237 | 0.084054 | 11α-methoxysaikosaponin f |
| CH15 | 13.48683 | 0.75203 | 11α-methoxysaikosaponin f_qt |
| CH16 | 21.50057 | 0.071379 | 3'-O-Acetylsaikosaponin D |
| CH17 | 26.17339 | 0.63219 | 3'-O-Acetylsaikosaponin D_qt |
| CH18 | 65.93409 | 0.091043 | 3',6'-O,O-diacetylsaikosaponin b2 |
| CH19 | 14.17098 | 0.73728 | 3',6'-O,O-diacetylsaikosaponin b2_qt |
| CH2 | 27.80866 | 0.076155 | Scopoletol |
| CH20 | 7.705255 | 0.10798 | 3'-O-acetylsaikosaponin b2 |
| CH21 | 13.88066 | 0.33458 | FLAVONE,3,5,7-TRIHYDROXY-3,4-DIMETHOXY |
| CH22 | 55.14803 | 0.41394 | Areapillin |
| CH23 | 41.60351 | 0.062144 | Ayapanin |
| CH24 | 29.94953 | 0.53015 | Longikaurin A |
| CH25 | 14.46272 | 0.74887 | Longispinogenin |
| CH26 | 47.82225 | 0.27864 | Octalupine |
| CH27 | 17.58733 | 0.21583 | 7,8,4'-Trihydroxyisoflavone |
| CH28 | 25.87557 | 0.63197 | Saikogenin F |
| CH29 | 51.8394 | 0.63197 | Saikogenin G |
| CH3 | 10.47032 | 0.27525 | quercetin |
| CH30 | 12.49626 | 0.086885 | Saikosaponin A |
| CH31 | 16.81125 | 0.13152 | Saikosaponin B |
| CH32 | 9.561587 | 0.086879 | Saikosaponin D |
| CH33 | 2.94037 | 0.055382 | Saikosaponin K |
| CH34 | 3.232958 | 0.026924 | Saikosaponin V |
| CH35 | 1.734283 | 0.078885 | Saikosaponin f |
| CH36 | 12.07869 | 0.74762 | 6,7,8-Trimethoxycoumarin |
| CH37 | 34.46355 | 0.12811 | Saikosaponin t |
| CH38 | 16.68668 | 0.71426 | Saikosaponin t_qt |
| CH39 | 81.60749 | 0.23333 | Sainfuran |
| CH4 | 36.89011 | 0.036518 | eugenol |
| CH40 | 43.16284 | 0.40714 | Thymonin |
| CH41 | 26.18846 | 0.091466 | fraxetin |
| CH42 | 2.478169 | 0.72495 | kaempferol-7-O-rhamnoside |
| CH43 | 3.571881 | 0.72499 | kaempferol-7-O-α-L-rhamnoside |
| CH44 | 36.66823 | 0.086466 | Limetin |
| CH45 | 5.283078 | 0.13146 | saikosaponin b1 |
| CH46 | 13.42641 | 0.73762 | saikosaponin b1_qt |
| CH47 | 9.55074 | 0.13147 | saikosaponin b2 |
| CH48 | 12.90001 | 0.73784 | saikosaponin b2_qt |
| CH49 | 4.431921 | 0.12222 | saikosaponin b3 |
| CH5 | 43.28127 | 0.029778 | o-Thymol |
| CH50 | 18.91821 | 0.69426 | saikosaponin b3_qt |
| CH51 | 4.527912 | 0.12221 | saikosaponin b4 |
| CH52 | 18.91821 | 0.6943 | saikosaponin b4_qt |
| CH53 | 5.120731 | 0.051481 | saikosaponin c |
| CH54 | 30.51828 | 0.63193 | saikosaponin c_qt |
| CH55 | 12.40966 | 0.088479 | saikosaponin e |
| CH56 | 17.62179 | 0.65736 | saikosaponin e_qt |
| CH57 | 42.97937 | 0.75693 | α-spinasterol |
| CH59 | 49.59981 | 0.010092 | Furfuranol |
| CH6 | 2.39849 | 0.30601 | Isorhamnetin |
| CH60 | 57.12813 | 0.6398 | Cubebin |
| CH7 | 3.201533 | 0.68282 | rutin |
| CH8 | 43.82985 | 0.75664 | Stigmasterol |
| CH9 | 31.85664 | 0.30712 | petunidi |
| HQ1 | 35.02838 | 0.71579 | campesterol |
| HQ11 | 22.99429 | 0.7761 | epiberberine |
| HQ12 | 27.25587 | 0.26546 | 5,8,2'-Trihydroxy-7-methoxyflavone |
| HQ13 | 40.2819 | 0.24188 | Carthamidin |
| HQ14 | 41.53938 | 0.20722 | Dihydrobaicalin_qt |
| HQ15 | 53.87782 | 0.33279 | Salvigenin |
| HQ16 | 93.43294 | 0.37375 | Ganhuangenin |
| HQ17 | 14.12928 | 0.37378 | Viscidulin III |
| HQ19 | 35.42827 | 0.24383 | 5,7,2',6'-Tetrahydroxyflavon |
| HQ2 | 40.44827 | 0.20723 | Norwogonin |
| HQ20 | 38.85886 | 0.22988 | dihydrooroxylin A |
| HQ21 | 125.0547 | 0.43789 | Skullcapflavone II |
| HQ23 | 18.68487 | 0.2915 | Panicolin |
| HQ24 | 34.76242 | 0.26666 | 5,7,4'-Trihydroxy-8-methoxyflavone |
| HQ25 | 12.56927 | 0.77131 | Baicalin |
| HQ27 | 29.49443 | 0.22598 | 11-Eicosenoic acid |
| HQ28 | 14.27086 | 0.3657 | Methyl lignocerate |
| HQ29 | 39.27534 | 0.22891 | 11,13-Eicosadienoic acid |
| HQ3 | 30.07322 | 0.35463 | 5,2'-Dihydroxy-6,7,8-trimethoxyflavone |
| HQ30 | 25.02246 | 0.2444 | 2',3',5,7-tetrahydroxyflavone |
| HQ31 | 37.00241 | 0.26833 | 5,7,4'-trihydroxy-6-methoxyflavanone |
| HQ32 | 38.39282 | 0.36629 | 5,2'-dihydroxy-7,8,6'-trimethoxyflavone |
| HQ34 | 13.6817 | 0.43456 | METHYL HEXACOSANOATE |
| HQ35 | 2.941386 | 0.62498 | Acteoside |
| HQ36 | 48.03082 | 0.1814 | Chrysin |
| HQ37 | 12.68937 | 0.66921 | Darendoside A |
| HQ38 | 6.360775 | 0.59206 | Darendoside B |
| HQ39 | 46.37778 | 0.23057 | Dihydrooroxylin A |
| HQ4 | 30.40885 | 0.85647 | coptisine |
| HQ41 | 13.98363 | 0.56188 | Isomartynoside |
| HQ42 | 4.174678 | 0.60622 | LEUCOSCEPTOSIDE A |
| HQ43 | 45.40775 | 0.23231 | Oroxylin A |
| HQ44 | 43.74214 | 0.36628 | Rivularin |
| HQ45 | 7.010258 | 0.19803 | Salidroside |
| HQ46 | 51.70113 | 0.29148 | Skullcapflavone I |
| HQ47 | 43.90662 | 0.43793 | SkulleapflavoneII |
| HQ48 | 32.7748 | 0.35463 | Tenaxin I |
| HQ5 | 33.54594 | 0.42162 | Supraene |
| HQ6 | 22.60737 | 0.18141 | chrysin |
| HQ7 | 21.09162 | 0.20888 | baicalein |
| HQ8 | 18.63631 | 0.24387 | scutellarein |
| HQ9 | 10.21151 | 0.75263 | Baicalin |
|  | | | |

We have used the yellow background to marking the ingredients which were selected in our next step research.

Table S2 the biological targets of compounds

| Targets Compounds ID | |
| --- | --- |
|  |  |
| ADCY5 | CH22 |
| ALDH2 | CH22 |
| ALOX5 | CH22 |
| CCR6 | CH22 |
| DYRK1A | CH22 |
| GDA | CH22 |
| HTR2A | CH22 |
| MAOB | CH22 |
| NEU | CH22 |
| ODC1 | CH22 |
| PDGFRB | CH22 |
| PLA2G2A | CH22 |
| TNFRSF1A | CH22 |
| TUBB1 | CH22 |
| TUBB2B | CH22 |
| ADRA2A | CH26 |
| CHRM2 | CH26 |
| CHRNA4 | CH26 |
| CHRNA7 | CH26 |
| CHRND | CH26 |
| DHCR7 | CH26 |
| EBP | CH26 |
| HRH1 | CH26 |
| HTR3A | CH26 |
| MAP2K4 | CH26 |
| OPRK1 | CH26 |
| SIGMAR1 | CH26 |
| SLC18A3 | CH26 |
| SRD5A1 | CH26 |
| SV2A | CH26 |
| CNR2 | CH29 |
| CYP27B1 | CH29 |
| DHCR7 | CH29 |
| DUOX2 | CH29 |
| ERG | CH29 |
| GAA | CH29 |
| GLB1 | CH29 |
| HSD11B2 | CH29 |
| MAN2A1 | CH29 |
| NDUFAB1 | CH29 |
| NR1i1 | CH29 |
| NR3C1 | CH29 |
| PYGM | CH29 |
| SFRP1 | CH29 |
| SLC10A2 | CH29 |
| SRD5A1 | CH29 |
| UGT2B7 | CH29 |
| APP | CH39 |
| ATP2A1 | CH39 |
| CYP2A5 | CH39 |
| CYP2A6 | CH39 |
| ESRRA | CH39 |
| HPGD | CH39 |
| MAOB | CH39 |
| NEU | CH39 |
| TUBB1 | CH39 |
| TUBB2B | CH39 |
| ADCY5 | CH40 |
| ALOX12 | CH40 |
| ALOX5 | CH40 |
| CYP1A1 | CH40 |
| NEU | CH40 |
| ODC1 | CH40 |
| PDGFRB | CH40 |
| TNFRSF1A | CH40 |
| TRAV4 | CH40 |
| TUBB1 | CH40 |
| TUBB2B | CH40 |
| CNR2 | CH54 |
| CYP27B1 | CH54 |
| DHCR7 | CH54 |
| DUOX2 | CH54 |
| ERG | CH54 |
| GAA | CH54 |
| GLB1 | CH54 |
| HSD11B2 | CH54 |
| MAN2A1 | CH54 |
| NDUFAB1 | CH54 |
| NR1i1 | CH54 |
| NR3C1 | CH54 |
| POLB | CH54 |
| PYGM | CH54 |
| SFRP1 | CH54 |
| SLC10A2 | CH54 |
| SRD5A1 | CH54 |
| UGT2B7 | CH54 |
| AR | CH57 |
| ATP1A1 | CH57 |
| CYP24A1 | CH57 |
| CYP27B1 | CH57 |
| DHCR7 | CH57 |
| DUOX2 | CH57 |
| EBP | CH57 |
| ELOVL6 | CH57 |
| HSD11B2 | CH57 |
| NDUFAB1 | CH57 |
| NR1i1 | CH57 |
| NR2B1 | CH57 |
| NR3C1 | CH57 |
| NR3C2 | CH57 |
| OPRM1 | CH57 |
| SLC10A1 | CH57 |
| SRD5A1 | CH57 |
| SRD5A2 | CH57 |
| UGT2B7 | CH57 |
| CYP2D6 | CH60 |
| HTR2A | CH60 |
| MAP2K4 | CH60 |
| STS | CH60 |
| TUBB2B | CH60 |
| APP | CH8 |
| AR | CH8 |
| ATP1A1 | CH8 |
| CYP24A1 | CH8 |
| CYP27B1 | CH8 |
| DHCR7 | CH8 |
| DUOX2 | CH8 |
| EBP | CH8 |
| ELOVL6 | CH8 |
| HSD11B2 | CH8 |
| NDUFAB1 | CH8 |
| NR1i1 | CH8 |
| NR2B1 | CH8 |
| NR3C1 | CH8 |
| NR3C2 | CH8 |
| OPRM1 | CH8 |
| SLC10A1 | CH8 |
| SRD5A1 | CH8 |
| SRD5A2 | CH8 |
| UGT2B7 | CH8 |
| APP | HQ1 |
| AR | HQ1 |
| CYP27B1 | HQ1 |
| DGAT-1 | HQ1 |
| DHCR7 | HQ1 |
| DUOX2 | HQ1 |
| EBP | HQ1 |
| HSD11B2 | HQ1 |
| KCNQ1 | HQ1 |
| NDUFAB1 | HQ1 |
| NR1i1 | HQ1 |
| NR2B1 | HQ1 |
| NR3C1 | HQ1 |
| NR3C2 | HQ1 |
| SLC10A1 | HQ1 |
| SRD5A1 | HQ1 |
| SRD5A2 | HQ1 |
| UGT2B7 | HQ1 |
| ADCY5 | HQ13 |
| ALOX12 | HQ13 |
| ALOX5 | HQ13 |
| ALPPL2 | HQ13 |
| CBR1 | HQ13 |
| CYP2A5 | HQ13 |
| HSD17B1 | HQ13 |
| HTR2A | HQ13 |
| KDM4D | HQ13 |
| MAP2K4 | HQ13 |
| NEU | HQ13 |
| PLA2G2A | HQ13 |
| SMAD3 | HQ13 |
| STS | HQ13 |
| TUBB2B | HQ13 |
| ADCY5 | HQ14 |
| ALOX12 | HQ14 |
| ALOX5 | HQ14 |
| ALPPL2 | HQ14 |
| ATP2A1 | HQ14 |
| CBR1 | HQ14 |
| CYP2A5 | HQ14 |
| HSD17B1 | HQ14 |
| HTR2A | HQ14 |
| KDM4D | HQ14 |
| NET | HQ14 |
| NEU | HQ14 |
| PLA2G2A | HQ14 |
| STS | HQ14 |
| TUBB2B | HQ14 |
| ADCY5 | HQ15 |
| ALDH2 | HQ15 |
| CCR6 | HQ15 |
| CYP1A1 | HQ15 |
| DYRK1A | HQ15 |
| GDA | HQ15 |
| HTR2A | HQ15 |
| MAOB | HQ15 |
| MTNR1A | HQ15 |
| NEU | HQ15 |
| ODC1 | HQ15 |
| PDGFRB | HQ15 |
| PRF-1 | HQ15 |
| TNFRSF1A | HQ15 |
| TUBA | HQ15 |
| TUBB1 | HQ15 |
| TUBB2B | HQ15 |
| ADCY5 | HQ16 |
| ALDH2 | HQ16 |
| ALOX5 | HQ16 |
| CBR1 | HQ16 |
| CYP1A1 | HQ16 |
| HTR2A | HQ16 |
| MAP2K4 | HQ16 |
| NEU | HQ16 |
| PDGFRB | HQ16 |
| PLA2G2A | HQ16 |
| SMAD3 | HQ16 |
| TNFRSF1A | HQ16 |
| TUBB1 | HQ16 |
| YARS | HQ16 |
| ADCY5 | HQ19 |
| ALDH2 | HQ19 |
| ALOX5 | HQ19 |
| CBR1 | HQ19 |
| CYP1A1 | HQ19 |
| CYP2A5 | HQ19 |
| HSD17B1 | HQ19 |
| HSD17B3 | HQ19 |
| KDM4D | HQ19 |
| MAP2K4 | HQ19 |
| MIF | HQ19 |
| NEU | HQ19 |
| PLA2G2A | HQ19 |
| SMAD3 | HQ19 |
| TLR4 | HQ19 |
| TNFRSF1A | HQ19 |
| TUBB2B | HQ19 |
| ADCY5 | HQ2 |
| ALDH2 | HQ2 |
| ALOX12 | HQ2 |
| ALOX5 | HQ2 |
| ALPPL2 | HQ2 |
| ATP2A1 | HQ2 |
| CBR1 | HQ2 |
| CYP2A5 | HQ2 |
| DHODH | HQ2 |
| DYRK1A | HQ2 |
| GDA | HQ2 |
| HSD17B1 | HQ2 |
| HSD17B3 | HQ2 |
| HTR2A | HQ2 |
| KDM4D | HQ2 |
| MAP2K4 | HQ2 |
| NEU | HQ2 |
| PLA2G2A | HQ2 |
| SMAD3 | HQ2 |
| TNFRSF1A | HQ2 |
| TUBB2B | HQ2 |
| ADCY5 | HQ24 |
| ALDH2 | HQ24 |
| ALOX12 | HQ24 |
| ALOX5 | HQ24 |
| ALPPL2 | HQ24 |
| ATP2A1 | HQ24 |
| CBR1 | HQ24 |
| CCR6 | HQ24 |
| CYP2A5 | HQ24 |
| DYRK1A | HQ24 |
| GDA | HQ24 |
| HSD17B1 | HQ24 |
| HTR2A | HQ24 |
| MAOB | HQ24 |
| MAP2K4 | HQ24 |
| NEU | HQ24 |
| NOS1 | HQ24 |
| PLA2G2A | HQ24 |
| PRF-1 | HQ24 |
| TNFRSF1A | HQ24 |
| TUBB1 | HQ24 |
| TUBB2B | HQ24 |
| AMD1 | HQ29 |
| AOC3 | HQ29 |
| BHMT | HQ29 |
| CNR2 | HQ29 |
| CPB2 | HQ29 |
| DNM1 | HQ29 |
| DUOX2 | HQ29 |
| EBP | HQ29 |
| ENPEP | HQ29 |
| GABBR1 | HQ29 |
| HPSE | HQ29 |
| HRH3 | HQ29 |
| KDM2A | HQ29 |
| LPAR3 | HQ29 |
| ND1 | HQ29 |
| NDUFAB1 | HQ29 |
| NOS1 | HQ29 |
| NR1i1 | HQ29 |
| PHF8 | HQ29 |
| PLA2G10 | HQ29 |
| PLA2G4B | HQ29 |
| POLB | HQ29 |
| SPHK1 | HQ29 |
| SPHK2 | HQ29 |
| SV2A | HQ29 |
| ADCY5 | HQ3 |
| ALOX5 | HQ3 |
| CBR1 | HQ3 |
| CYP1A1 | HQ3 |
| HTR2A | HQ3 |
| NEU | HQ3 |
| ODC1 | HQ3 |
| PDGFRB | HQ3 |
| TNFRSF1A | HQ3 |
| TUBB1 | HQ3 |
| ADCY5 | HQ31 |
| ALOX12 | HQ31 |
| ALOX5 | HQ31 |
| ALPPL2 | HQ31 |
| ATP2A1 | HQ31 |
| CBR1 | HQ31 |
| ESRRA | HQ31 |
| GRIN2B | HQ31 |
| HSD17B1 | HQ31 |
| HTR2A | HQ31 |
| KDM4D | HQ31 |
| NEU | HQ31 |
| PLA2G2A | HQ31 |
| STS | HQ31 |
| TUBB2B | HQ31 |
| ADCY5 | HQ32 |
| ALDH2 | HQ32 |
| ALOX5 | HQ32 |
| CBR1 | HQ32 |
| CYP1A1 | HQ32 |
| HTR2A | HQ32 |
| MAP2K4 | HQ32 |
| MIF | HQ32 |
| NEU | HQ32 |
| ODC1 | HQ32 |
| PDGFRB | HQ32 |
| PLA2G2A | HQ32 |
| TLR4 | HQ32 |
| TNFRSF1A | HQ32 |
| TUBB1 | HQ32 |
| TUBB2B | HQ32 |
| ABCG2 | HQ36 |
| ADCY5 | HQ36 |
| ALDH2 | HQ36 |
| ALOX12 | HQ36 |
| ALOX5 | HQ36 |
| ALPPL2 | HQ36 |
| ATP2A1 | HQ36 |
| CA2 | HQ36 |
| CBR1 | HQ36 |
| CDK5 | HQ36 |
| CDK6 | HQ36 |
| CYP1A1 | HQ36 |
| CYP1B1 | HQ36 |
| CYP2A5 | HQ36 |
| CYP2A6 | HQ36 |
| CYP2B6 | HQ36 |
| CYP2C9 | HQ36 |
| DHODH | HQ36 |
| FLT3 | HQ36 |
| GSK3B | HQ36 |
| HSD17B1 | HQ36 |
| HSD17B3 | HQ36 |
| HSP90AA1 | HQ36 |
| HTR2A | HQ36 |
| KDM4D | HQ36 |
| MAOB | HQ36 |
| MAP2K4 | HQ36 |
| MAPK10 | HQ36 |
| MAPK14 | HQ36 |
| NET | HQ36 |
| NEU | HQ36 |
| NOX4 | HQ36 |
| PIM1 | HQ36 |
| PLA2G2A | HQ36 |
| SMAD3 | HQ36 |
| TLR4 | HQ36 |
| TNKS2 | HQ36 |
| TRAV4 | HQ36 |
| TUBB2B | HQ36 |
| ADCY5 | HQ39 |
| ALPPL2 | HQ39 |
| ATP2A1 | HQ39 |
| CBR1 | HQ39 |
| CYP2A5 | HQ39 |
| HSD17B1 | HQ39 |
| HTR2A | HQ39 |
| KDM4D | HQ39 |
| MTNR1A | HQ39 |
| NET | HQ39 |
| NEU | HQ39 |
| PLA2G2A | HQ39 |
| STS | HQ39 |
| TUBB2B | HQ39 |
| ADCY5 | HQ4 |
| BMP4 | HQ4 |
| CYP2A5 | HQ4 |
| HRH3 | HQ4 |
| HTR2C | HQ4 |
| PDE2A | HQ4 |
| STS | HQ4 |
| TUBB1 | HQ4 |
| TUBB2B | HQ4 |
| WEE1 | HQ4 |
| ADCY5 | HQ43 |
| ALDH2 | HQ43 |
| ALOX12 | HQ43 |
| ALOX5 | HQ43 |
| ALPPL2 | HQ43 |
| ATP2A1 | HQ43 |
| CYP1A1 | HQ43 |
| CYP2A5 | HQ43 |
| ESRRA | HQ43 |
| HSD17B1 | HQ43 |
| HSD17B3 | HQ43 |
| HTR2A | HQ43 |
| KDM4D | HQ43 |
| MAP2K4 | HQ43 |
| MTNR1A | HQ43 |
| NEU | HQ43 |
| NOX4 | HQ43 |
| ODC1 | HQ43 |
| PI3K | HQ43 |
| PLA2G2A | HQ43 |
| TNFRSF1A | HQ43 |
| TRAV4 | HQ43 |
| TUBB1 | HQ43 |
| TUBB2B | HQ43 |
| ADCY5 | HQ44 |
| ALDH2 | HQ44 |
| ALOX5 | HQ44 |
| CBR1 | HQ44 |
| CYP1A1 | HQ44 |
| HTR2A | HQ44 |
| MAP2K4 | HQ44 |
| MIF | HQ44 |
| NEU | HQ44 |
| NOX4 | HQ44 |
| ODC1 | HQ44 |
| PDGFRB | HQ44 |
| PI3K | HQ44 |
| PLA2G2A | HQ44 |
| SMAD3 | HQ44 |
| TNFRSF1A | HQ44 |
| TRAV4 | HQ44 |
| TUBB1 | HQ44 |
| TUBB2B | HQ44 |
| ADCY5 | HQ46 |
| ALDH2 | HQ46 |
| ALOX5 | HQ46 |
| ALPPL2 | HQ46 |
| CBR1 | HQ46 |
| CYP1A1 | HQ46 |
| CYP2A5 | HQ46 |
| HSD17B1 | HQ46 |
| HTR2A | HQ46 |
| MAP2K4 | HQ46 |
| MIF | HQ46 |
| NEU | HQ46 |
| NOX4 | HQ46 |
| ODC1 | HQ46 |
| PDGFRB | HQ46 |
| PI3K | HQ46 |
| PLA2G2A | HQ46 |
| SMAD3 | HQ46 |
| STS | HQ46 |
| TNFRSF1A | HQ46 |
| TRAV4 | HQ46 |
| TUBB1 | HQ46 |
| TUBB2B | HQ46 |
| ADCY5 | HQ47 |
| ALDH2 | HQ47 |
| CBR1 | HQ47 |
| MAP2K4 | HQ47 |
| NEU | HQ47 |
| NOX4 | HQ47 |
| ODC1 | HQ47 |
| PDGFRB | HQ47 |
| PLA2G2A | HQ47 |
| TNFRSF1A | HQ47 |
| TRAV4 | HQ47 |
| TUBB1 | HQ47 |
| ADCY5 | HQ48 |
| ALDH2 | HQ48 |
| ALOX5 | HQ48 |
| CBR1 | HQ48 |
| CYP1A1 | HQ48 |
| HTR2A | HQ48 |
| MAP2K4 | HQ48 |
| NEU | HQ48 |
| NOX4 | HQ48 |
| ODC1 | HQ48 |
| PDGFRB | HQ48 |
| PI3K | HQ48 |
| TNFRSF1A | HQ48 |
| TRAV4 | HQ48 |
| TUBB1 | HQ48 |
| CHRND | HQ5 |
| CPB2 | HQ5 |
| DNM1 | HQ5 |
| DUOX2 | HQ5 |
| EBP | HQ5 |
| ENPEP | HQ5 |
| HPSE | HQ5 |
| HRH3 | HQ5 |
| LPAR3 | HQ5 |
| NDUFAB1 | HQ5 |
| NOS1 | HQ5 |
| NR1i1 | HQ5 |
| PHF8 | HQ5 |
| PLA2G4B | HQ5 |
| SPHK1 | HQ5 |
| SPHK2 | HQ5 |
|  |  |

Table s3 GO biological classification of the targets

| GO biological process | Gene count | P-Value | Associate Gene ID |
| --- | --- | --- | --- |
| regulation of MAP kinase activity | 15 | 6.86E-09 | ADRA2A,BMP4,CHRNA7,ERBB2,FLT3,HTR2A,LPAR3,MAP2K4,MAPK10,MAPK14,MIF,NOX4,PDGFRB,SFRP1,TLR4 |
| cellular response to steroid hormone stimulus | 11 | 1.23E-06 | AR,ATP1A1,BMP4,CYP27B1,ESRRA,FLT3,NR3C1,NR3C2,RXRA,SFRP1,SRD5A1 |
| arachidonic acid metabolic process | 10 | 1.32E-12 | ALOX12,CBR1,CYP1A1,CYP1B1,CYP2A6,CYP2B6,CYP2C9,CYP2D6,PLA2G10,PLA2G4B |
| steroid biosynthetic process | 10 | 1.85E-07 | ATP1A1,CYP27B1,DHCR7,EBP,GLB1,HSD11B2,HSD17B1,HSD17B3,SRD5A1,SRD5A2 |
| reactive oxygen species biosynthetic process | 9 | 2.4E-08 | CYP1A1,CYP1B1,DUOX2,HSP90AA1,MAOB,NOS1,OPRM1,SMAD3,TLR4 |
| monoamine transport | 9 | 1.51E-09 | ADRA2A,CHRNA4,CHRNA7,HRH3,HTR2A,MAOB,NOS1,OPRK1,SLC18A3 |
| phospholipase C-activating G-protein coupled receptor signaling pathway | 9 | 2.4E-08 | ADRA2A,CHRM2,HRH1,HRH3,HTR2A,HTR2C,LPAR3,OPRK1,OPRM1 |
| positive regulation of ERK1 and ERK2 cascade | 9 | 4.25E-06 | BMP4,HTR2A,HTR2C,MIF,NOX4,OPRM1,PDGFRB,PLA2G2A,TLR4 |
| regulation of reactive oxygen species metabolic process | 8 | 1.94E-05 | CYP1B1,HSP90AA1,MAPK14,NOX4,OPRM1,PDGFRB,SMAD3,TLR4 |
| steroid hormone mediated signaling pathway | 8 | 5.28E-05 | AR,BMP4,CYP27B1,ESRRA,NR3C1,NR3C2,RXRA,SFRP1 |
| activation of MAPK activity | 7 | 0.000102 | ADRA2A,CHRNA7,LPAR3,MAP2K4,MAPK10,MAPK14,TLR4 |
| synaptic transmission, cholinergic | 6 | 6.13E-07 | CHRM2,CHRNA4,CHRNA7,CHRND,HRH3,HTR3A |
| hormone biosynthetic process | 6 | 1.28E-05 | ATP1A1,DUOX2,HSD17B1,HSD17B3,SRD5A1,SRD5A2 |
| nitric oxide biosynthetic process | 6 | 1.18E-05 | CYP1B1,HSP90AA1,NOS1,OPRM1,SMAD3,TLR4 |
| sensory perception of pain | 6 | 6.59E-05 | CDK5,CHRNA4,CNR2,HTR2A,OPRK1,OPRM1 |
| sterol metabolic process | 6 | 0.000578 | APP,CYP1B1,DHCR7,EBP,GLB1,RXRA |
| androgen metabolic process | 4 | 3.85E-05 | HSD17B3,SRD5A1,SRD5A2,UGT2B7 |
| cGMP metabolic process | 4 | 0.000482 | HTR2C,MTNR1A,NOS1,PDE2A |
| positive regulation of nitric oxide biosynthetic process | 4 | 0.000279 | HSP90AA1,OPRM1,SMAD3,TLR4 |
| prostaglandin metabolic process | 4 | 0.000106 | CBR1,HPGD,MIF,TNFRSF1A |
| steroid catabolic process | 4 | 1.17E-05 | CYP24A1,CYP27B1,SRD5A2,STS |
| androgen biosynthetic process | 3 | 6.69E-05 | HSD17B3,SRD5A1,SRD5A2 |
| norepinephrine secretion | 3 | 0.000284 | ADRA2A,CHRNA7,HRH3 |
| opioid receptor signaling pathway | 3 | 3.69E-05 | OPRK1,OPRM1,SIGMAR1 |

Table S4 the results of MeSH analysis

| Gene Name | Disease | MesH |
| --- | --- | --- |
| TNFRSF1A | Multiple Sclerosis | Autoimmune Diseases of the Nervous System |
| OPRM1 | Hypotension, Orthostatic | Autonomic Nervous System Diseases |
| DHODH | Bacterial infections | Bacterial Infections and Mycoses |
| MAPK14 | Endotoxemia | Bacterial Infections and Mycoses |
| ALOX5 | Endotoxemia | Bacterial Infections and Mycoses |
| DHODH | Fungal diseases | Bacterial Infections and Mycoses |
| AOC3 | Paratuberculosis | Bacterial Infections and Mycoses |
| GSK3B | Peritonitis | Bacterial Infections and Mycoses |
| TLR4 | Sepsis | Bacterial Infections and Mycoses |
| MIF | Sepsis | Bacterial Infections and Mycoses |
| MIF | Septic shock | Bacterial Infections and Mycoses |
| CYP2C9 | Tuberculosis | Bacterial Infections and Mycoses |
| STS | Breast cancer | Breast Neoplasms |
| CYP1B1 | Breast cancer | Breast Neoplasms |
| ESRRA | Breast cancer | Breast Neoplasms |
| HSP90AA1 | Breast cancer | Breast Neoplasms |
| CYP1B1 | Breast Neoplasms | Breast Neoplasms |
| MIF | Breast Neoplasms | Breast Neoplasms |
| CYP1A1 | Breast Neoplasms | Breast Neoplasms |
| ESRRA | Breast Neoplasms | Breast Neoplasms |
| SFRP1 | Breast Neoplasms | Breast Neoplasms |
| HPSE | Breast Neoplasms | Breast Neoplasms |
| CNR2 | Breast Neoplasms | Breast Neoplasms |
| HSP90AA1 | Breast Neoplasms | Breast Neoplasms |
| PDE2A | Breast Neoplasms | Breast Neoplasms |
| AR | Breast Neoplasms | Breast Neoplasms |
| BMP4 | Breast Neoplasms | Breast Neoplasms |
| PIM1 | Breast Neoplasms | Breast Neoplasms |
| CYP2B6 | Breast Neoplasms | Breast Neoplasms |
| CYP2D6 | Breast Neoplasms | Breast Neoplasms |
| CBR1 | Breast Neoplasms | Breast Neoplasms |
| CYP2A6 | Breast Neoplasms | Breast Neoplasms |
| ABCG2 | Breast Neoplasms | Breast Neoplasms |
| CYP2C9 | Cardiovascular Diseases | Cardiovascular Diseases |
| HTR2A | Alzheimer Disease | Central Nervous System Diseases |
| CHRNA7 | Alzheimer Disease | Central Nervous System Diseases |
| CYP2D6 | Alzheimer Disease | Central Nervous System Diseases |
| GSK3B | Alzheimer Disease | Central Nervous System Diseases |
| APP | Alzheimer Disease | Central Nervous System Diseases |
| SIGMAR1 | Amyotrophic Lateral Sclerosis | Central Nervous System Diseases |
| CYP2D6 | Basal Ganglia Diseases | Central Nervous System Diseases |
| HTR2A | Basal Ganglia Diseases | Central Nervous System Diseases |
| APP | Brain Diseases | Central Nervous System Diseases |
| CYP2A6 | Brain Diseases | Central Nervous System Diseases |
| CHRNA7 | Brain Injuries | Central Nervous System Diseases |
| TLR4 | Brain Injuries | Central Nervous System Diseases |
| TNFRSF1A | Brain Injuries | Central Nervous System Diseases |
| CHRM2 | Brain Injuries | Central Nervous System Diseases |
| TNFRSF1A | Brain Ischemia | Central Nervous System Diseases |
| HRH3 | Central nervous system diseases | Central Nervous System Diseases |
| NOS1 | Cerebellar Diseases | Central Nervous System Diseases |
| APP | Cerebral Amyloid Angiopathy | Central Nervous System Diseases |
| ALOX5 | Cerebral vasospasm | Central Nervous System Diseases |
| GRIN2B | Convulsions | Central Nervous System Diseases |
| GRIN2B | Dementia | Central Nervous System Diseases |
| APP | Dementia | Central Nervous System Diseases |
| GRIN2B | Drug-induced dyskinesias | Central Nervous System Diseases |
| HTR2A | Dyskinesias | Central Nervous System Diseases |
| OPRM1 | Dyskinesias | Central Nervous System Diseases |
| CYP1A1 | Epilepsy | Central Nervous System Diseases |
| CYP2C9 | Epilepsy | Central Nervous System Diseases |
| ABCG2 | Epilepsy | Central Nervous System Diseases |
| CYP2A6 | Epilepsy | Central Nervous System Diseases |
| SV2A | Epilepsy | Central Nervous System Diseases |
| UGT2B7 | Epilepsy | Central Nervous System Diseases |
| ADRA2A | Epilepsy | Central Nervous System Diseases |
| CHRM2 | Epilepsy | Central Nervous System Diseases |
| HRH1 | Epilepsy | Central Nervous System Diseases |
| GRIN2B | Epilepsy | Central Nervous System Diseases |
| OPRM1 | Epilepsy | Central Nervous System Diseases |
| CHRNA4 | Epilepsy, Frontal Lobe | Central Nervous System Diseases |
| CHRNA4 | Epilepsy, Frontal Lobe | Central Nervous System Diseases |
| CHRNA7 | Epilepsy, Generalized | Central Nervous System Diseases |
| CYP2D6 | Epilepsy, Tonic-Clonic | Central Nervous System Diseases |
| SIGMAR1 | Frontotemporal Lobar Degeneration | Central Nervous System Diseases |
| GLB1 | Gangliosidosis, GM1 | Central Nervous System Diseases |
| GAA | Glycogen Storage Disease Type II | Central Nervous System Diseases |
| GLB1 | GM1 gangliosidosis | Central Nervous System Diseases |
| ALOX5 | Infarction, Middle Cerebral Artery | Central Nervous System Diseases |
| HTR2A | Migraine | Central Nervous System Diseases |
| HTR2A | Migraine Disorders | Central Nervous System Diseases |
| OPRM1 | Movement Disorders | Central Nervous System Diseases |
| CYP2D6 | Parkinson Disease | Central Nervous System Diseases |
| MAOB | Parkinson Disease | Central Nervous System Diseases |
| MAOB | Parkinson's disease | Central Nervous System Diseases |
| GRIN2B | Parkinson's disease | Central Nervous System Diseases |
| ALOX5 | Prion diseases | Central Nervous System Diseases |
| NOS1 | Seizures | Central Nervous System Diseases |
| SIGMAR1 | Seizures | Central Nervous System Diseases |
| CHRNA7 | Seizures | Central Nervous System Diseases |
| CHRNA4 | Seizures | Central Nervous System Diseases |
| OPRK1 | Seizures | Central Nervous System Diseases |
| OPRM1 | Seizures | Central Nervous System Diseases |
| CYP1A1 | Seizures | Central Nervous System Diseases |
| APP | Spinal Cord Diseases | Central Nervous System Diseases |
| AR | Spinal muscular atrophy (SMA) | Central Nervous System Diseases |
| NOS1 | Status Epilepticus | Central Nervous System Diseases |
| GRIN2B | Stroke | Central Nervous System Diseases |
| CYP2C9 | Stroke | Central Nervous System Diseases |
| HTR2A | tardive dyskinesia | Central Nervous System Diseases |
| CYP2D6 | tardive dyskinesia | Central Nervous System Diseases |
| GSK3B | Abnormalities, Multiple | Congenital, Hereditary, and Neonatal Diseases and Abnormalities |
| GLB1 | Dermatitis, Atopic | Congenital, Hereditary, and Neonatal Diseases and Abnormalities |
| PYGM | Glycogen Storage Disease Type V | Congenital, Hereditary, and Neonatal Diseases and Abnormalities |
| CHRNA7 | Hernia, Umbilical | Congenital, Hereditary, and Neonatal Diseases and Abnormalities |
| STS | Ichthyosis, X-Linked | Congenital, Hereditary, and Neonatal Diseases and Abnormalities |
| HSD11B2 | Mineralocorticoid Excess Syndrome, Apparent | Congenital, Hereditary, and Neonatal Diseases and Abnormalities |
| GLB1 | Mucopolysaccharidosis IV | Congenital, Hereditary, and Neonatal Diseases and Abnormalities |
| ALOX5 | Sjogren-Larsson syndrome | Congenital, Hereditary, and Neonatal Diseases and Abnormalities |
| DHCR7 | Smith-Lemli-Opitz Syndrome | Congenital, Hereditary, and Neonatal Diseases and Abnormalities |
| ND1 | Leber optic atrophy | Cranial Nerve Diseases |
| ND1 | Optic Atrophy, Hereditary, Leber | Cranial Nerve Diseases |
| CYP1A1 | Hamartoma | Cysts |
| MAPK14 | Cholestasis | Digestive System Diseases |
| ALOX5 | Colitis | Digestive System Diseases |
| SPHK1 | Colitis, Ulcerative | Digestive System Diseases |
| CCR6 | Colitis, Ulcerative | Digestive System Diseases |
| TLR4 | Crohn Disease | Digestive System Diseases |
| SMAD3 | Crohn Disease | Digestive System Diseases |
| MAPK14 | Crohn's disease, unspecified | Digestive System Diseases |
| SLC10A2 | Crohn's disease, unspecified | Digestive System Diseases |
| TNFRSF1A | Cystic Fibrosis | Digestive System Diseases |
| CYP2C9 | Drug-Induced Liver Injury | Digestive System Diseases |
| CYP2A6 | Drug-Induced Liver Injury | Digestive System Diseases |
| CYP2A5 | Drug-Induced Liver Injury | Digestive System Diseases |
| NOS1 | End Stage Liver Disease | Digestive System Diseases |
| TLR4 | Enterocolitis, Necrotizing | Digestive System Diseases |
| ALDH2 | Enterocolitis, Necrotizing | Digestive System Diseases |
| STS | Fatty Liver | Digestive System Diseases |
| TLR4 | Fatty Liver | Digestive System Diseases |
| OPRM1 | Hepatic Encephalopathy | Digestive System Diseases |
| MAOB | Hepatic Encephalopathy | Digestive System Diseases |
| NOS1 | Hepatic Encephalopathy | Digestive System Diseases |
| CYP2B6 | Hepatic Veno-Occlusive Disease | Digestive System Diseases |
| SFRP1 | Inflammatory Bowel Diseases | Digestive System Diseases |
| NOS1 | Intestinal Perforation | Digestive System Diseases |
| HTR3A | Irritable bowel syndrome | Digestive System Diseases |
| CNR2 | Liver Cirrhosis | Digestive System Diseases |
| SMAD3 | Liver Cirrhosis | Digestive System Diseases |
| DHCR7 | Liver Cirrhosis | Digestive System Diseases |
| HSD11B2 | Liver Cirrhosis | Digestive System Diseases |
| SMAD3 | Liver Cirrhosis, Alcoholic | Digestive System Diseases |
| ABCG2 | Liver Cirrhosis, Biliary | Digestive System Diseases |
| TNFRSF1A | Liver Cirrhosis, Biliary | Digestive System Diseases |
| PDGFRB | Liver Cirrhosis, Experimental | Digestive System Diseases |
| ALDH2 | Liver Cirrhosis, Experimental | Digestive System Diseases |
| SLC10A1 | Liver Cirrhosis, Experimental | Digestive System Diseases |
| HPSE | Liver Cirrhosis, Experimental | Digestive System Diseases |
| CNR2 | Pancreatitis | Digestive System Diseases |
| CYP2C9 | Peptic Ulcer Hemorrhage | Digestive System Diseases |
| ALOX5 | Ulcerative colitis | Digestive System Diseases |
| MIF | Ulcerative colitis | Digestive System Diseases |
| MAOB | Colonic Neoplasms | Digestive System Neoplasms |
| CBR1 | Colonic Neoplasms | Digestive System Neoplasms |
| ALOX5 | Colonic Neoplasms | Digestive System Neoplasms |
| ODC1 | Colonic Neoplasms | Digestive System Neoplasms |
| NOX4 | Colonic Neoplasms | Digestive System Neoplasms |
| PDE2A | Colorectal cancer | Digestive System Neoplasms |
| PLA2G2A | Colorectal Neoplasms | Digestive System Neoplasms |
| ALDH2 | Colorectal Neoplasms | Digestive System Neoplasms |
| SFRP1 | Colorectal Neoplasms | Digestive System Neoplasms |
| CYP2A6 | Colorectal Neoplasms | Digestive System Neoplasms |
| ABCG2 | Colorectal Neoplasms | Digestive System Neoplasms |
| CYP1B1 | Colorectal Neoplasms | Digestive System Neoplasms |
| BMP4 | Colorectal Neoplasms | Digestive System Neoplasms |
| TLR4 | Colorectal Neoplasms | Digestive System Neoplasms |
| ODC1 | Colorectal Neoplasms | Digestive System Neoplasms |
| ALDH2 | Esophageal Neoplasms | Digestive System Neoplasms |
| ALOX5 | Gastrointestinal cancers | Digestive System Neoplasms |
| MAPK14 | Liver Neoplasms | Digestive System Neoplasms |
| ALOX5 | Pancreatic cancer | Digestive System Neoplasms |
| CA2 | Pancreatic cancer | Digestive System Neoplasms |
| APP | Pancreatic cancer | Digestive System Neoplasms |
| CNR2 | Pancreatic Neoplasms | Digestive System Neoplasms |
| ABCG2 | Pancreatic Neoplasms | Digestive System Neoplasms |
| CYP1B1 | Pancreatic Neoplasms | Digestive System Neoplasms |
| ALOX5 | Stomach Neoplasms | Digestive System Neoplasms |
| CA2 | Stomach Neoplasms | Digestive System Neoplasms |
| CYP2A6 | Stomach Neoplasms | Digestive System Neoplasms |
| HSP90AA1 | Ovarian cancer | Endocrine Gland Neoplasms |
| CYP1B1 | Ovarian Neoplasms | Endocrine Gland Neoplasms |
| TLR4 | Ovarian Neoplasms | Endocrine Gland Neoplasms |
| HPGD | Thyroid Neoplasms | Endocrine Gland Neoplasms |
| ALDH2 | Diabetes Complications | Endocrine System Diseases |
| ATP1A1 | Hyperaldosteronism | Endocrine System Diseases |
| GLB1 | Corneal Opacity | Eye Diseases |
| CA2 | Glaucoma | Eye Diseases |
| TLR4 | Macular Degeneration | Eye Diseases |
| TLR4 | Macular Degeneration | Eye Diseases |
| NR3C1 | Ocular Hypertension | Eye Diseases |
| MTNR1A | Retinal Diseases | Eye Diseases |
| CYP24A1 | Abortion, Spontaneous | Female Urogenital Diseases and Pregnancy Complications |
| CYP1A1 | Abortion, Spontaneous | Female Urogenital Diseases and Pregnancy Complications |
| HSD17B1 | Abortion, Spontaneous | Female Urogenital Diseases and Pregnancy Complications |
| ALPPL2 | Abortion, Spontaneous | Female Urogenital Diseases and Pregnancy Complications |
| BHMT | Abruptio Placentae | Female Urogenital Diseases and Pregnancy Complications |
| AR | Diabetes, Gestational | Female Urogenital Diseases and Pregnancy Complications |
| NR3C2 | Endometriosis | Female Urogenital Diseases and Pregnancy Complications |
| SRD5A1 | Endometriosis | Female Urogenital Diseases and Pregnancy Complications |
| SRD5A2 | Endometriosis | Female Urogenital Diseases and Pregnancy Complications |
| PLA2G2A | Endometriosis | Female Urogenital Diseases and Pregnancy Complications |
| MAOB | Endometriosis | Female Urogenital Diseases and Pregnancy Complications |
| NR3C1 | Endometriosis | Female Urogenital Diseases and Pregnancy Complications |
| HSD17B1 | Endometriosis | Female Urogenital Diseases and Pregnancy Complications |
| NOS1 | Fetal Alcohol Spectrum Disorders | Female Urogenital Diseases and Pregnancy Complications |
| AR | Infertility, Female | Female Urogenital Diseases and Pregnancy Complications |
| HSD11B2 | Pregnancy Complications | Female Urogenital Diseases and Pregnancy Complications |
| SIGMAR1 | Prenatal Exposure Delayed Effects | Female Urogenital Diseases and Pregnancy Complications |
| CYP2D6 | Prenatal Exposure Delayed Effects | Female Urogenital Diseases and Pregnancy Complications |
| CYP1B1 | Mouth Neoplasms | Head and Neck Neoplasms |
| CYP2A6 | Nasopharyngeal Neoplasms | Head and Neck Neoplasms |
| CYP2D6 | Acute Coronary Syndrome | Heart Diseases |
| TLR4 | Acute Coronary Syndrome | Heart Diseases |
| CYP2B6 | Acute Coronary Syndrome | Heart Diseases |
| CYP2C9 | Acute Coronary Syndrome | Heart Diseases |
| TUBB1 | Acute Coronary Syndrome | Heart Diseases |
| OPRK1 | Arrhythmias, Cardiac | Heart Diseases |
| CYP2C9 | Atrial Fibrillation | Heart Diseases |
| CHRM2 | Bradycardia | Heart Diseases |
| CYP2D6 | Bradycardia | Heart Diseases |
| KCNQ1 | Cardiac arrhythmias | Heart Diseases |
| CYP1B1 | Cardiomegaly | Heart Diseases |
| CYP1A1 | Cardiomegaly | Heart Diseases |
| GSK3B | Cardiomegaly | Heart Diseases |
| ADRA2A | Cardiomegaly | Heart Diseases |
| MAPK14 | Cardiomyopathies | Heart Diseases |
| ADCY5 | Cardiomyopathies | Heart Diseases |
| CBR1 | Cardiomyopathies | Heart Diseases |
| CYP2C9 | Cardiomyopathies | Heart Diseases |
| NR3C2 | Cardiomyopathy, Dilated | Heart Diseases |
| GAA | Cardiomyopathy, Hypertrophic | Heart Diseases |
| TLR4 | Coronary Artery Disease | Heart Diseases |
| CYP2C9 | Coronary Artery Disease | Heart Diseases |
| ALOX5 | Coronary Artery Disease | Heart Diseases |
| PLA2G2A | Coronary Artery Disease | Heart Diseases |
| CYP24A1 | Coronary Artery Disease | Heart Diseases |
| ADRA2A | Heart Diseases | Heart Diseases |
| NOX4 | Heart Failure | Heart Diseases |
| ADRA2A | Heart Failure | Heart Diseases |
| GSK3B | Heart Failure | Heart Diseases |
| ATP2A1 | Heart Failure | Heart Diseases |
| NR3C2 | Heart Failure | Heart Diseases |
| TNFRSF1A | Heart Failure | Heart Diseases |
| ATP1A1 | Heart Failure | Heart Diseases |
| GAA | Hypertrophy, Left Ventricular | Heart Diseases |
| ADRA2A | Ischemic heart disease | Heart Diseases |
| KCNQ1 | Jervell-Lange Nielsen Syndrome | Heart Diseases |
| KCNQ1 | Long QT Syndrome | Heart Diseases |
| NR3C2 | Myocardial Infarction | Heart Diseases |
| CYP2C9 | Myocardial Infarction | Heart Diseases |
| GSK3B | Myocardial Infarction | Heart Diseases |
| HSD11B2 | Myocardial Infarction | Heart Diseases |
| ABCG2 | Myocardial Infarction | Heart Diseases |
| MAPK14 | Myocardial Ischemia | Heart Diseases |
| ATP1A1 | Myocardial Ischemia | Heart Diseases |
| PLA2G2A | Myocardial Ischemia | Heart Diseases |
| KCNQ1 | Romano-Ward Syndrome | Heart Diseases |
| ALDH2 | Ventricular Dysfunction | Heart Diseases |
| GAA | Ventricular Dysfunction | Heart Diseases |
| NR3C2 | Ventricular Dysfunction, Left | Heart Diseases |
| HSP90AA1 | Hematological Malignancies | Hematologic Neoplasms |
| MAOB | Anemia, Megaloblastic | Hemic and Lymphatic Diseases |
| CYP2D6 | Anemia, Sickle Cell | Hemic and Lymphatic Diseases |
| DHODH | Anemia, Sickle Cell | Hemic and Lymphatic Diseases |
| UGT2B7 | Anemia, Sickle Cell | Hemic and Lymphatic Diseases |
| DHODH | beta-Thalassemia | Hemic and Lymphatic Diseases |
| SPHK1 | Myelodysplastic Syndromes | Hemic and Lymphatic Diseases |
| CYP2A6 | Neutropenia | Hemic and Lymphatic Diseases |
| DHODH | Spherocytosis, Hereditary | Hemic and Lymphatic Diseases |
| DHODH | Autoimmune Diseases | Immune System Diseases |
| TNFRSF1A | Autoimmune Diseases | Immune System Diseases |
| FLT3 | Autoimmune Diseases | Immune System Diseases |
| CYP2C9 | Drug Hypersensitivity | Immune System Diseases |
| TNFRSF1A | Hypersensitivity | Immune System Diseases |
| CYP1A1 | Hypersensitivity | Immune System Diseases |
| CCR6 | Hypersensitivity | Immune System Diseases |
| CNR2 | Immune System Diseases | Immune System Diseases |
| FLT3 | Acute myeloid leukemia | Leukemia |
| SPHK1 | Leukemia | Leukemia |
| AR | Leukemia | Leukemia |
| ERG | Leukemia | Leukemia |
| DHODH | Leukemia, Erythroblastic, Acute | Leukemia |
| POLB | Leukemia, Lymphocytic, Chronic, B-Cell | Leukemia |
| MAPK14 | Leukemia, Myelogenous, Chronic, BCR-ABL Positive | Leukemia |
| ALOX5 | Leukemia, Myelogenous, Chronic, BCR-ABL Positive | Leukemia |
| CNR2 | Leukemia, Myeloid, Acute | Leukemia |
| ERG | Leukemia, Myeloid, Acute | Leukemia |
| CDK6 | Leukemia, Myeloid, Acute | Leukemia |
| FLT3 | Leukemia, Myeloid, Acute | Leukemia |
| FLT3 | Leukemia, Promyelocytic, Acute | Leukemia |
| CDK6 | Precursor Cell Lymphoblastic Leukemia-Lymphoma | Leukemia |
| CYP1B1 | Precursor Cell Lymphoblastic Leukemia-Lymphoma | Leukemia |
| ABCG2 | Precursor T-Cell Lymphoblastic Leukemia-Lymphoma | Leukemia |
| HSP90AA1 | Lymphoma, Large-Cell, Anaplastic | Lymphoma |
| GSK3B | Lymphoma, Mantle-Cell | Lymphoma |
| BHMT | Lymphoma, Non-Hodgkin | Lymphoma |
| CYP2D6 | Acute Kidney Injury | Male Urogenital Diseases |
| GSK3B | Acute Kidney Injury | Male Urogenital Diseases |
| CYP2C9 | Acute Kidney Injury | Male Urogenital Diseases |
| AR | Androgen-Insensitivity Syndrome | Male Urogenital Diseases |
| CYP2B6 | Cystitis | Male Urogenital Diseases |
| AOC3 | Diabetic Nephropathies | Male Urogenital Diseases |
| HSD17B3 | Disorders of Sex Development | Male Urogenital Diseases |
| PDE2A | Erectile dysfunction | Male Urogenital Diseases |
| MIF | Glomerulonephritis | Male Urogenital Diseases |
| ALOX5 | Glomerulonephritis | Male Urogenital Diseases |
| TLR4 | Glomerulonephritis, IGA | Male Urogenital Diseases |
| ELOVL6 | Glomerulonephritis, IGA | Male Urogenital Diseases |
| HPSE | Glomerulonephritis, IGA | Male Urogenital Diseases |
| GAA | Glomerulonephritis, Membranous | Male Urogenital Diseases |
| AR | Infertility, Male | Male Urogenital Diseases |
| CYP1A1 | Infertility, Male | Male Urogenital Diseases |
| ALDH2 | Infertility, Male | Male Urogenital Diseases |
| CYP2B6 | Kidney Failure, Chronic | Male Urogenital Diseases |
| CYP2D6 | Kidney Failure, Chronic | Male Urogenital Diseases |
| NOX4 | Kidney Failure, Chronic | Male Urogenital Diseases |
| CYP2C9 | Nephritis, Interstitial | Male Urogenital Diseases |
| CYP2D6 | Nephritis, Interstitial | Male Urogenital Diseases |
| HPSE | Nephrosis | Male Urogenital Diseases |
| ALOX5 | Nephrosis | Male Urogenital Diseases |
| HSD11B2 | Nephrotic Syndrome | Male Urogenital Diseases |
| SRD5A2 | Prostatic Hyperplasia | Male Urogenital Diseases |
| NR3C2 | Pseudohypoaldosteronism | Male Urogenital Diseases |
| PIM1 | Pyelonephritis | Male Urogenital Diseases |
| CA2 | Renal failure | Male Urogenital Diseases |
| CYP24A1 | Renal Insufficiency | Male Urogenital Diseases |
| NOS1 | Urination Disorders | Male Urogenital Diseases |
| GSK3B | Arthritis, Experimental | Musculoskeletal Diseases |
| TLR4 | Arthritis, Rheumatoid | Musculoskeletal Diseases |
| DHODH | Arthritis, Rheumatoid | Musculoskeletal Diseases |
| ABCG2 | Arthritis, Rheumatoid | Musculoskeletal Diseases |
| CDK6 | Arthritis, Rheumatoid | Musculoskeletal Diseases |
| ALOX5 | Arthritis, Rheumatoid | Musculoskeletal Diseases |
| CCR6 | Arthritis, Rheumatoid | Musculoskeletal Diseases |
| MIF | Arthritis, Rheumatoid | Musculoskeletal Diseases |
| GLB1 | Bone Diseases, Developmental | Musculoskeletal Diseases |
| DHCR7 | Bone Diseases, Metabolic | Musculoskeletal Diseases |
| DUOX2 | Congenital Hypothyroidism | Musculoskeletal Diseases |
| SMAD3 | Craniofacial Abnormalities | Musculoskeletal Diseases |
| SMAD3 | Loeys-Dietz Syndrome | Musculoskeletal Diseases |
| ND1 | MELAS Syndrome | Musculoskeletal Diseases |
| CYP2D6 | Muscle Rigidity | Musculoskeletal Diseases |
| PYGM | Muscular Diseases | Musculoskeletal Diseases |
| SMAD3 | Osteoarthritis | Musculoskeletal Diseases |
| ALOX5 | Osteoarthritis | Musculoskeletal Diseases |
| HPGD | Osteoarthropathy, Primary Hypertrophic | Musculoskeletal Diseases |
| GLB1 | Osteochondrodysplasias | Musculoskeletal Diseases |
| ADCY5 | Osteoporosis | Musculoskeletal Diseases |
| CA2 | Osteoporosis | Musculoskeletal Diseases |
| CYP27B1 | Polymyositis | Musculoskeletal Diseases |
| HPGD | Primary hypertrophic osteoarthropathy (PHO) | Musculoskeletal Diseases |
| MAPK14 | Rheumatoid arthritis, unspecified | Musculoskeletal Diseases |
| DHODH | Rheumatoid arthritis, unspecified | Musculoskeletal Diseases |
| MIF | Rheumatoid arthritis, unspecified | Musculoskeletal Diseases |
| CYP27B1 | Rickets | Musculoskeletal Diseases |
| AR | Spinal and bulbar muscular atrophy | Musculoskeletal Diseases |
| WEE1 | Cancer | Neoplasms |
| CDK6 | Cancer, unspecific | Neoplasms |
| MIF | Cancer, unspecific | Neoplasms |
| DHODH | Cancer, unspecific | Neoplasms |
| AMD1 | Cancer, unspecific | Neoplasms |
| CYP2A6 | Neoplasms | Neoplasms |
| ABCG2 | Neoplasms | Neoplasms |
| CBR1 | Neoplasms | Neoplasms |
| STS | Tumors | Neoplasms |
| DHODH | Tumors | Neoplasms |
| HSD11B2 | Tumors | Neoplasms |
| PDGFRB | Ewing's sarcoma | Neoplasms, Connective and Soft Tissue |
| ERG | Ewing's sarcoma | Neoplasms, Connective and Soft Tissue |
| SMAD3 | Leiomyoma | Neoplasms, Connective and Soft Tissue |
| SFRP1 | Leiomyoma | Neoplasms, Connective and Soft Tissue |
| HPGD | Carcinoma, Medullary | Neoplasms, Germ Cell and Embryonal |
| CDK6 | Glioblastoma | Neoplasms, Germ Cell and Embryonal |
| PIM1 | Glioblastoma | Neoplasms, Germ Cell and Embryonal |
| PDGFRB | Glioma | Neoplasms, Germ Cell and Embryonal |
| CYP2B6 | Gliosarcoma | Neoplasms, Germ Cell and Embryonal |
| CDK6 | Medulloblastoma | Neoplasms, Germ Cell and Embryonal |
| CYP1B1 | Melanoma | Neoplasms, Germ Cell and Embryonal |
| HSP90AA1 | Melanoma | Neoplasms, Germ Cell and Embryonal |
| TLR4 | Melanoma | Neoplasms, Germ Cell and Embryonal |
| ALOX12 | Melanoma | Neoplasms, Germ Cell and Embryonal |
| HPSE | Melanoma | Neoplasms, Germ Cell and Embryonal |
| MAOB | Pheochromocytoma | Neoplasms, Germ Cell and Embryonal |
| BMP4 | ACTH-Secreting Pituitary Adenoma | Neoplasms, Glandular and Epithelial |
| CA2 | Adenocarcinoma | Neoplasms, Glandular and Epithelial |
| CYP2A6 | Adenocarcinoma | Neoplasms, Glandular and Epithelial |
| ATP1A1 | Adenoma | Neoplasms, Glandular and Epithelial |
| ALOX5 | Adenoma | Neoplasms, Glandular and Epithelial |
| ABCG2 | Adenoma | Neoplasms, Glandular and Epithelial |
| CYP1B1 | Adenoma | Neoplasms, Glandular and Epithelial |
| ALOX5 | Bronchiolar carcinoma | Neoplasms, Glandular and Epithelial |
| TLR4 | Carcinoma | Neoplasms, Glandular and Epithelial |
| ADCY5 | Carcinoma | Neoplasms, Glandular and Epithelial |
| MIF | Carcinoma | Neoplasms, Glandular and Epithelial |
| TUBB2B | Carcinoma | Neoplasms, Glandular and Epithelial |
| GDA | Carcinoma | Neoplasms, Glandular and Epithelial |
| CA2 | Carcinoma, Hepatocellular | Neoplasms, Glandular and Epithelial |
| CYP1A1 | Carcinoma, Hepatocellular | Neoplasms, Glandular and Epithelial |
| CBR1 | Carcinoma, Hepatocellular | Neoplasms, Glandular and Epithelial |
| AR | Carcinoma, Hepatocellular | Neoplasms, Glandular and Epithelial |
| CYP2A6 | Carcinoma, Large Cell | Neoplasms, Glandular and Epithelial |
| CYP2A6 | Carcinoma, Small Cell | Neoplasms, Glandular and Epithelial |
| CYP1B1 | Carcinoma, Squamous Cell | Neoplasms, Glandular and Epithelial |
| DHODH | Hepatocellular carcinoma | Neoplasms, Glandular and Epithelial |
| MAPK14 | Multiple Myeloma | Neoplasms, Glandular and Epithelial |
| HSP90AA1 | Multiple Myeloma | Neoplasms, Glandular and Epithelial |
| ODC1 | Papilloma | Neoplasms, Glandular and Epithelial |
| BMP4 | Prolactinoma | Neoplasms, Glandular and Epithelial |
| AR | Prostatic Intraepithelial Neoplasia | Neoplasms, Glandular and Epithelial |
| PIM1 | Prostatic Intraepithelial Neoplasia | Neoplasms, Glandular and Epithelial |
| HPSE | Neoplasm Invasiveness | Neoplastic Processes |
| TLR4 | Neoplasm Invasiveness | Neoplastic Processes |
| CHRNA7 | Neoplasm Invasiveness | Neoplastic Processes |
| ALOX5 | Neoplasm Invasiveness | Neoplastic Processes |
| AR | Neoplasm Recurrence, Local | Neoplastic Processes |
| NOS1 | Nervous System Diseases | Nervous System Diseases |
| ABCG2 | Nervous System Diseases | Nervous System Diseases |
| CYP2C9 | Nervous System Diseases | Nervous System Diseases |
| CHRNA4 | Nervous System Diseases | Nervous System Diseases |
| SLC10A2 | Nervous System Diseases | Nervous System Diseases |
| TUBB2B | Malformations of Cortical Development | Nervous System Malformations |
| BHMT | Neural Tube Defects | Nervous System Malformations |
| TUBB2B | Polymicrogyria | Nervous System Malformations |
| CHRNA7 | Spina Bifida Cystica | Nervous System Malformations |
| CYP2C9 | Brain Neoplasms | Nervous System Neoplasms |
| CDK6 | Brain Stem Neoplasms | Nervous System Neoplasms |
| CHRNA4 | Neurodegenerative diseases | Neurodegenerative Diseases |
| OPRK1 | Neurodegenerative diseases | Neurodegenerative Diseases |
| APP | Neurodegenerative diseases | Neurodegenerative Diseases |
| SIGMAR1 | Amnesia | Neurologic Manifestations |
| HRH3 | Amnesia | Neurologic Manifestations |
| APP | Amnesia | Neurologic Manifestations |
| TLR4 | Angina, Stable | Neurologic Manifestations |
| GRIN2B | Chronic pain | Neurologic Manifestations |
| CYP2D6 | Coma | Neurologic Manifestations |
| CHRNA4 | Dystonia | Neurologic Manifestations |
| CYP2D6 | Dystonia | Neurologic Manifestations |
| APP | Fragile X Syndrome | Neurologic Manifestations |
| ALOX5 | Hyperalgesia | Neurologic Manifestations |
| GRIN2B | Hyperalgesia | Neurologic Manifestations |
| HTR2A | Hyperalgesia | Neurologic Manifestations |
| CNR2 | Hyperalgesia | Neurologic Manifestations |
| ALOX12 | Hyperalgesia | Neurologic Manifestations |
| NOS1 | Hyperalgesia | Neurologic Manifestations |
| OPRM1 | Hyperalgesia | Neurologic Manifestations |
| GABBR1 | Hyperkinesis | Neurologic Manifestations |
| HTR2A | Hyperkinesis | Neurologic Manifestations |
| HTR2C | Hyperkinesis | Neurologic Manifestations |
| GRIN2B | Intellectual Disability | Neurologic Manifestations |
| APP | Learning Disorders | Neurologic Manifestations |
| SIGMAR1 | Learning Disorders | Neurologic Manifestations |
| APP | Memory Disorders | Neurologic Manifestations |
| SIGMAR1 | Memory Disorders | Neurologic Manifestations |
| MIF | Memory Disorders | Neurologic Manifestations |
| HRH3 | Memory Disorders | Neurologic Manifestations |
| CHRNA7 | Memory Disorders | Neurologic Manifestations |
| GSK3B | Muscular Atrophy | Neurologic Manifestations |
| OPRM1 | Neuralgia | Neurologic Manifestations |
| NOS1 | Neurobehavioral Manifestations | Neurologic Manifestations |
| CHRNA7 | Neurobehavioral Manifestations | Neurologic Manifestations |
| CHRM2 | Neurogenic bladder | Neurologic Manifestations |
| APP | Neurogenic Inflammation | Neurologic Manifestations |
| HTR2C | neuropathic pain | Neurologic Manifestations |
| CHRNA4 | Pain | Neurologic Manifestations |
| OPRK1 | Pain | Neurologic Manifestations |
| HRH1 | Pain | Neurologic Manifestations |
| CHRNA7 | Pain | Neurologic Manifestations |
| OPRM1 | Pain, Postoperative | Neurologic Manifestations |
| APP | Paralysis | Neurologic Manifestations |
| CHRND | Paralysis | Neurologic Manifestations |
| CYP2D6 | Tremor | Neurologic Manifestations |
| CHRM2 | Urinary Bladder, Neurogenic | Neurologic Manifestations |
| CHRND | Congenital myasthenic syndrome | Neuromuscular Diseases |
| CDK5 | Peripheral Nervous System Diseases | Neuromuscular Diseases |
| POLB | Peripheral Nervous System Diseases | Neuromuscular Diseases |
| CYP24A1 | Peripheral Nervous System Diseases | Neuromuscular Diseases |
| SRD5A1 | Peripheral Nervous System Diseases | Neuromuscular Diseases |
| UGT2B7 | Peripheral Nervous System Diseases | Neuromuscular Diseases |
| ELOVL6 | Peripheral Nervous System Diseases | Neuromuscular Diseases |
| SRD5A2 | Peripheral Nervous System Diseases | Neuromuscular Diseases |
| TLR4 | Peripheral Nervous System Diseases | Neuromuscular Diseases |
| ALOX12 | Peripheral Nervous System Diseases | Neuromuscular Diseases |
| SLC10A2 | Peripheral Nervous System Diseases | Neuromuscular Diseases |
| CYP2D6 | Manganese Poisoning | Neurotoxicity Syndromes |
| ESRRA | Diabetes mellitus | Nutritional and Metabolic Diseases |
| ESRRA | Diabetes Mellitus, Experimental | Nutritional and Metabolic Diseases |
| STS | Diabetes Mellitus, Experimental | Nutritional and Metabolic Diseases |
| CHRM2 | Diabetes Mellitus, Experimental | Nutritional and Metabolic Diseases |
| CYP1A1 | Diabetes Mellitus, Experimental | Nutritional and Metabolic Diseases |
| TNFRSF1A | Diabetes Mellitus, Experimental | Nutritional and Metabolic Diseases |
| ND1 | Diabetes Mellitus, Experimental | Nutritional and Metabolic Diseases |
| AOC3 | Diabetes Mellitus, Experimental | Nutritional and Metabolic Diseases |
| HSD11B2 | Diabetes Mellitus, Type 1 | Nutritional and Metabolic Diseases |
| NOS1 | Diabetes Mellitus, Type 1 | Nutritional and Metabolic Diseases |
| CHRM2 | Diabetes Mellitus, Type 1 | Nutritional and Metabolic Diseases |
| KCNQ1 | Diabetes Mellitus, Type 2 | Nutritional and Metabolic Diseases |
| CYP2C9 | Diabetes Mellitus, Type 2 | Nutritional and Metabolic Diseases |
| ADCY5 | Diabetes Mellitus, Type 2 | Nutritional and Metabolic Diseases |
| TNFRSF1A | Diabetes Mellitus, Type 2 | Nutritional and Metabolic Diseases |
| BHMT | Diabetes Mellitus, Type 2 | Nutritional and Metabolic Diseases |
| CYP27B1 | Hypercalcemia | Nutritional and Metabolic Diseases |
| HTR2C | Hypercholesterolemia | Nutritional and Metabolic Diseases |
| HTR2A | Hypercholesterolemia | Nutritional and Metabolic Diseases |
| HTR3A | Hypercholesterolemia | Nutritional and Metabolic Diseases |
| ABCG2 | Hypercholesterolemia | Nutritional and Metabolic Diseases |
| NR3C1 | Hypokalemia | Nutritional and Metabolic Diseases |
| STS | Insulin Resistance | Nutritional and Metabolic Diseases |
| MAPK14 | Insulin Resistance | Nutritional and Metabolic Diseases |
| AR | Insulin Resistance | Nutritional and Metabolic Diseases |
| ESRRA | Metabolic Diseases | Nutritional and Metabolic Diseases |
| HTR2A | Metabolic Diseases | Nutritional and Metabolic Diseases |
| HTR2C | Metabolic Syndrome X | Nutritional and Metabolic Diseases |
| PYGM | Noninsulin-dependent diabetes mellitus | Nutritional and Metabolic Diseases |
| GSK3B | Noninsulin-dependent diabetes mellitus | Nutritional and Metabolic Diseases |
| HTR2C | Obesity | Nutritional and Metabolic Diseases |
| CPB2 | Obesity | Nutritional and Metabolic Diseases |
| STS | Obesity | Nutritional and Metabolic Diseases |
| ESRRA | Obesity | Nutritional and Metabolic Diseases |
| HTR2A | Obesity | Nutritional and Metabolic Diseases |
| NR3C1 | Type 2 Diabetes | Nutritional and Metabolic Diseases |
| BMP4 | Vitamin A Deficiency | Nutritional and Metabolic Diseases |
| CHRNA7 | Auditory Perceptual Disorders | Otorhinolaryngologic Diseases |
| TLR4 | Hearing Loss | Otorhinolaryngologic Diseases |
| KCNQ1 | Hearing Loss, Noise-Induced | Otorhinolaryngologic Diseases |
| ATP1A1 | Vestibular Diseases | Otorhinolaryngologic Diseases |
| ODC1 | African trypanosomiasis | Parasitic Diseases |
| CYP2A6 | Liver Diseases, Parasitic | Parasitic Diseases |
| DHODH | Malaria | Parasitic Diseases |
| ODC1 | Malaria | Parasitic Diseases |
| CYP2A6 | Opisthorchiasis | Parasitic Diseases |
| AMD1 | Parasitic diseases | Parasitic Diseases |
| DHODH | Parasitic diseases | Parasitic Diseases |
| ALDH2 | Precancerous Conditions | Precancerous Conditions |
| MAPK14 | Adult respiratory distress syndrome | Respiratory Tract Diseases |
| ALOX5 | Adult respiratory distress syndrome | Respiratory Tract Diseases |
| ALOX5 | aspirin-induced asthma | Respiratory Tract Diseases |
| ALDH2 | Asthma | Respiratory Tract Diseases |
| HSD11B2 | Asthma | Respiratory Tract Diseases |
| ALOX5 | Asthma | Respiratory Tract Diseases |
| CHRM2 | Chronic obstructive pulmonary disease | Respiratory Tract Diseases |
| OPRM1 | Cough | Respiratory Tract Diseases |
| OPRM1 | Dyspnea | Respiratory Tract Diseases |
| OPRK1 | Dyspnea | Respiratory Tract Diseases |
| ALOX5 | Hypertension, Pulmonary | Respiratory Tract Diseases |
| NR3C1 | Lung Diseases, Obstructive | Respiratory Tract Diseases |
| TNFRSF1A | Lung Injury | Respiratory Tract Diseases |
| CYP1A1 | Lung Injury | Respiratory Tract Diseases |
| TLR4 | Lung Injury | Respiratory Tract Diseases |
| TNFRSF1A | Pneumonia | Respiratory Tract Diseases |
| CCR6 | Pneumonia | Respiratory Tract Diseases |
| HTR2A | Pulmonary Disease, Chronic Obstructive | Respiratory Tract Diseases |
| CYP1A1 | Pulmonary Disease, Chronic Obstructive | Respiratory Tract Diseases |
| CYP2C9 | Pulmonary Embolism | Respiratory Tract Diseases |
| CYP2A6 | Pulmonary Emphysema | Respiratory Tract Diseases |
| ALOX5 | Pulmonary fibrosis | Respiratory Tract Diseases |
| TLR4 | Respiratory Hypersensitivity | Respiratory Tract Diseases |
| HRH1 | Respiratory Hypersensitivity | Respiratory Tract Diseases |
| HRH1 | Rhinitis | Respiratory Tract Diseases |
| HRH1 | Seasonal allergic rhinitis | Respiratory Tract Diseases |
| SRD5A1 | Acne | Skin and Connective Tissue Diseases |
| AR | Alopecia | Skin and Connective Tissue Diseases |
| SRD5A2 | Alopecia | Skin and Connective Tissue Diseases |
| CDK6 | Chloracne | Skin and Connective Tissue Diseases |
| PIM1 | Dermatitis, Allergic Contact | Skin and Connective Tissue Diseases |
| CYP1A1 | Dermatitis, Allergic Contact | Skin and Connective Tissue Diseases |
| TLR4 | Dermatitis, Contact | Skin and Connective Tissue Diseases |
| ALDH2 | Dermatitis, Occupational | Skin and Connective Tissue Diseases |
| CYP1A1 | Drug Eruptions | Skin and Connective Tissue Diseases |
| ALOX5 | Drug Eruptions | Skin and Connective Tissue Diseases |
| SRD5A1 | Hirsutism | Skin and Connective Tissue Diseases |
| HRH1 | Pruritus | Skin and Connective Tissue Diseases |
| HTR3A | Pruritus | Skin and Connective Tissue Diseases |
| MAPK14 | Psoriasis | Skin and Connective Tissue Diseases |
| ABCG2 | Psoriasis | Skin and Connective Tissue Diseases |
| ALOX5 | Psoriasis | Skin and Connective Tissue Diseases |
| MAPK14 | Skin Diseases | Skin and Connective Tissue Diseases |
| ALOX12 | Skin Diseases | Skin and Connective Tissue Diseases |
| MIF | Systemic lupus erythematosus | Skin and Connective Tissue Diseases |
| ALOX5 | Urticaria | Skin and Connective Tissue Diseases |
| CCR6 | Vitiligo | Skin and Connective Tissue Diseases |
| ODC1 | Skin Neoplasms | Skin Neoplasms |
| CYP2D6 | Insomnia | Sleep Wake Disorders |
| MTNR1A | Insomnia | Sleep Wake Disorders |
| CHRNA4 | Sleep Disorders | Sleep Wake Disorders |
| CYP2B6 | Sleep Disorders | Sleep Wake Disorders |
| MIF | Stevens-Johnson Syndrome | Stomatognathic Diseases |
| ABCG2 | Carcinoma, Non-Small-Cell Lung | Thoracic Neoplasms |
| CYP2A6 | Carcinoma, Non-Small-Cell Lung | Thoracic Neoplasms |
| HPSE | Lung Cancer | Thoracic Neoplasms |
| CYP1B1 | Lung Neoplasms | Thoracic Neoplasms |
| TLR4 | Lung Neoplasms | Thoracic Neoplasms |
| CHRNA7 | Lung Neoplasms | Thoracic Neoplasms |
| MAPK14 | Lung Neoplasms | Thoracic Neoplasms |
| CYP2A6 | Lung Neoplasms | Thoracic Neoplasms |
| CYP24A1 | Lung Neoplasms | Thoracic Neoplasms |
| ABCG2 | Lung Neoplasms | Thoracic Neoplasms |
| HSP90AA1 | Non-small Cell Lung Cancer | Thoracic Neoplasms |
| NOS1 | Trauma, Nervous System | Trauma, Nervous System |
| CDK5 | Bladder cancer | Urogenital Neoplasms |
| ALOX5 | Cervical cancer | Urogenital Neoplasms |
| CYP1A1 | Endometrial Neoplasms | Urogenital Neoplasms |
| SRD5A2 | Endometrial Neoplasms | Urogenital Neoplasms |
| CYP1A1 | Kidney Neoplasms | Urogenital Neoplasms |
| SRD5A2 | Prostate cancer | Urogenital Neoplasms |
| HSP90AA1 | Prostate cancer | Urogenital Neoplasms |
| CYP1B1 | Prostate cancer | Urogenital Neoplasms |
| SPHK2 | Prostate cancer | Urogenital Neoplasms |
| SPHK1 | Prostate cancer | Urogenital Neoplasms |
| SRD5A1 | Prostate cancer | Urogenital Neoplasms |
| AR | Prostate cancer | Urogenital Neoplasms |
| HPSE | Prostate cancer | Urogenital Neoplasms |
| SRD5A2 | Prostatic Neoplasms | Urogenital Neoplasms |
| CYP1A1 | Prostatic Neoplasms | Urogenital Neoplasms |
| CYP2A6 | Prostatic Neoplasms | Urogenital Neoplasms |
| SLC10A2 | Prostatic Neoplasms | Urogenital Neoplasms |
| AR | Prostatic Neoplasms | Urogenital Neoplasms |
| HSD17B1 | Prostatic Neoplasms | Urogenital Neoplasms |
| ERG | Prostatic Neoplasms | Urogenital Neoplasms |
| NR3C1 | Prostatic Neoplasms | Urogenital Neoplasms |
| HSD17B3 | Prostatic Neoplasms | Urogenital Neoplasms |
| MIF | Prostatic Neoplasms | Urogenital Neoplasms |
| TLR4 | Prostatic Neoplasms | Urogenital Neoplasms |
| GSK3B | Prostatic Neoplasms | Urogenital Neoplasms |
| CYP1B1 | Prostatic Neoplasms | Urogenital Neoplasms |
| HPGD | Prostatic Neoplasms | Urogenital Neoplasms |
| CBR1 | Prostatic Neoplasms | Urogenital Neoplasms |
| SRD5A1 | Prostatic Neoplasms | Urogenital Neoplasms |
| POLB | Urinary Bladder Neoplasms | Urogenital Neoplasms |
| CYP1A1 | Urinary Bladder Neoplasms | Urogenital Neoplasms |
| UGT2B7 | Urinary Bladder Neoplasms | Urogenital Neoplasms |
| ALOX5 | Urological cancers | Urogenital Neoplasms |
| SMAD3 | Uterine Neoplasms | Urogenital Neoplasms |
| SMAD3 | Aneurysm, Dissecting | Vascular Diseases |
| SMAD3 | Aortic Aneurysm | Vascular Diseases |
| CYP1A1 | Arteritis | Vascular Diseases |
| CNR2 | Atherosclerosis | Vascular Diseases |
| TLR4 | Atherosclerosis | Vascular Diseases |
| PLA2G2A | Atherosclerosis | Vascular Diseases |
| ALOX5 | Atherosclerosis | Vascular Diseases |
| HRH1 | Atherosclerosis | Vascular Diseases |
| NR3C1 | Hypertension | Vascular Diseases |
| HRH3 | Hypertension | Vascular Diseases |
| ADRA2A | Hypertension | Vascular Diseases |
| HSD11B2 | Hypertension | Vascular Diseases |
| ENPEP | Hypertension | Vascular Diseases |
| AR | Hypertension | Vascular Diseases |
| CYP1A1 | Hypertension | Vascular Diseases |
| ADCY5 | Hypertension | Vascular Diseases |
| ATP1A1 | Hypertension | Vascular Diseases |
| HRH1 | Hypotension | Vascular Diseases |
| MAOB | Hypotension | Vascular Diseases |
| ADRA2A | Hypotension | Vascular Diseases |
| GRIN2B | Hypotension | Vascular Diseases |
| CYP1A1 | Peripheral Vascular Diseases | Vascular Diseases |
| PLA2G2A | Reperfusion Injury | Vascular Diseases |
| ALOX5 | Reperfusion Injury | Vascular Diseases |
| CYP2C9 | Thromboembolism | Vascular Diseases |
| FLT3 | Thrombosis | Vascular Diseases |
| CPB2 | Thrombosis | Vascular Diseases |
| MAPK14 | Thrombosis | Vascular Diseases |
| ALDH2 | Vascular Diseases | Vascular Diseases |
| CYP2C9 | Venous Thrombosis | Vascular Diseases |
| CYP2A6 | Hepatitis B | Virus Diseases |
| SLC10A1 | Hepatitis B | Virus Diseases |
| OPRM1 | Hepatitis C | Virus Diseases |
| CYP2A6 | Hepatitis C | Virus Diseases |
| SIGMAR1 | HIV Infections | Virus Diseases |
| CYP2A6 | HIV Infections | Virus Diseases |
| CYP2B6 | HIV Infections | Virus Diseases |
| UGT2B7 | HIV Infections | Virus Diseases |
| HSP90AA1 | HIV Infections | Virus Diseases |
| ODC1 | HIV Infections | Virus Diseases |
